# In silico transcriptome screens identify epidermal growth factor receptor inhibitors as therapeutics for noise-induced hearing loss

**DOI:** 10.1126/sciadv.adk2299

**Published:** 2024-06-19

**Authors:** Sarath Vijayakumar, Joseph A. DiGuiseppi, Parinaz Jila Dabestani, William G. Ryan, Rene Vielman Quevedo, Yuju Li, Jack Diers, Shu Tu, Jonathan Fleegel, Cassidy Nguyen, Lauren M. Rhoda, Ali Sajid Imami, Abdul-Rizaq Ali Hamoud, Sándor Lovas, Robert E. McCullumsmith, Marisa Zallocchi, Jian Zuo

**Affiliations:** ^1^ Department of Biomedical Sciences, School of Medicine, Creighton University, Omaha, NE 68178, USA.; ^2^ Department of Neurosciences_,_ University of Toledo, Toledo, OH 43614, USA_._; ^3^ Neurosciences Institute, ProMedica, Toledo, OH 43606, USA.; ^4^ Ting Therapeutics, University of California San Diego, 9310 Athena Circle, San Diego, CA 92037, USA.

## Abstract

Noise-induced hearing loss (NIHL) is a common sensorineural hearing impairment that lacks U.S. Food and Drug Administration–approved drugs. To fill the gap in effective screening models, we used an in silico transcriptome-based drug screening approach, identifying 22 biological pathways and 64 potential small molecule treatments for NIHL. Two of these, afatinib and zorifertinib [epidermal growth factor receptor (EGFR) inhibitors], showed efficacy in zebrafish and mouse models. Further tests with EGFR knockout mice and EGF-morpholino zebrafish confirmed their protective role against NIHL. Molecular studies in mice highlighted EGFR’s crucial involvement in NIHL and the protective effect of zorifertinib. When given orally, zorifertinib was found in the perilymph with favorable pharmacokinetics. In addition, zorifertinib combined with AZD5438 (a cyclin-dependent kinase 2 inhibitor) synergistically prevented NIHL in zebrafish. Our results underscore the potential for in silico transcriptome-based drug screening in diseases lacking efficient models and suggest EGFR inhibitors as potential treatments for NIHL, meriting clinical trials.

## Teaser

EGFR inhibitors protect against noise-induced hearing loss.

## INTRODUCTION

Noise-induced hearing loss (NIHL) is one of the most common forms of sensorineural hearing loss and occupational hazards in modern society worldwide (*1*–*6*). The impact of hearing loss on our society is such that one in five Americans may exhibit some degree of hearing loss (*7*, *8*). Occupational noise exposure has been attributed to about 16% of disabling hearing loss worldwide (*9*). Approximately 1 billion young adults and adolescents are at risk for NIHL due to recreational exposure to noise via personal audio systems, loud music in clubs, and at music concerts (World Health Organization, 2015). NIHL significantly affects the military and veterans. Centers for Disease Control and Prevention reported that military veterans have a four times higher risk of developing severe hearing loss compared to age- and occupation-matched civilians (*10*). NIHL has a negative impact on the quality of life and carries a substantial financial burden on affected individuals (*11*). The total economic cost of hearing loss, including NIHL, is more than $750 billion annually worldwide (*12*). Hearing loss has been implicated as a potential risk factor for accelerated cognitive decline and impairment in the increasingly socially isolated elderly (*13*–*15*). It has also been linked to the development of depression in some individuals (*13*). Despite the enormous societal impact, there are no drugs that are approved by the U.S. Food and Drug Administration (FDA) to prevent or recover NIHL. Currently, hearing aids that amplify the sound and cochlear implants are the mainstay approaches to treating hearing loss. Depending on the intensity and duration of the noise exposure, acoustic trauma can cause damage to the cochlear hair cells, supporting cells, hair cell synapses, spiral ganglion neurons, or other regions of the cochlea, all of which can lead to hearing impairment (*16*, *17*). Substantial advances have been made in our understanding of the cellular and molecular processes involved in the pathophysiology of NIHL, including but not limited to glutamate excitotoxicity, oxidative stress, imbalance of ions in the endolymph, inflammation, and microcirculation changes in the stria vascularis (*18*). Since NIHL is a predictable form of hearing loss, it is feasible to prevent it by inhibiting cochlear cell death or promoting cochlear cell survival. Despite extensive research in recent years, most candidate compounds currently in preclinical and clinical trials are related to antioxidants, vitamins, and glutathione metabolism, and their effectiveness remains unclear (*19*, *20*).

Over the past two decades, high-throughput screening (HTS) has become a standard approach in drug discovery. Recently, HTS has uncovered small molecule otoprotectant candidates (*21*–*27*). These chemical phenotypic screenings are unbiased in that they explore diverse biological pathways that prevent cisplatin- or antibiotic-induced cochlear cell death in cell lines, explants, or zebrafish models. Unfortunately, such drug screens for noise protection cannot be easily applied to cell lines, explants, or zebrafish models since these assays cannot accurately simulate the inner ear milieu during noise exposure for HTS. Thus, we used a computational drug discovery approach to identify the biological pathways and drugs of most interest to the prevention and treatment of NIHL. Drug development strategies based on transcriptomics are advantageous in that they do not require a large amount of a priori knowledge about particular diseases or drugs (*28*, *29*). In silico screening using the connectivity map (CMap) requires a gene expression profiling database of small molecules to be compared with the gene expression signatures of a disease or condition such as NIHL (*30*). The library of integrated network-based cellular signatures (LINCS) L1000 dataset currently has more than a million gene expression profiles in small molecule–treated cell lines (*31*). Drug candidates can be predicted by comparing the LINCS L1000CDS^2^ perturbation signatures and the disease-specific signatures extracted from the gene expression omnibus (*32*). By comparing published mouse cochlear gene expression profiles in NIHL and the LINCS L1000 dataset, we identified 22 candidate pathways and 64 candidate drugs protective against NIHL. Among the top hits are tyrosine kinase inhibitors that target epidermal growth factor receptor (EGFR) and human epidermal growth factor receptor 2 (HER2).

EGFR is a member of the epidermal growth factor receptor family that includes HER1 (erbB1 and EGFR), HER2 (erbB2 and NEU), HER3 (erbB3), and HER4 (erbB4), which activate and regulate diverse processes including cell survival, proliferation, differentiation, and migration (*33*). EGFR is widely expressed on the surface of mammalian epithelial, mesenchymal, and neuronal cells. EGFR transcripts have been detected in postnatal rat cochlear organotypic cultures, in multiple cell types in neonatal mouse cochleae, including both inner and outer hair cells (IHCs and OHCs respectively), spiral ganglion neurons, and Deiters’ and Hensen cells (*34*–*36*). ErbB2, ErbB3, and ErbB4 immunolabeling is present in the cochlear and vestibular sensory epithelia of adult chinchilla (*37*). While studies have looked at the role of ErbB2 in the survival and regeneration of hair cells in the cochlea (*38*, *39*), inhibition of EGFR as a therapeutic intervention for NIHL has not been investigated. In this study, we focused on providing “proof of concept” for the future drug development of EGFR inhibitors as otoprotective drugs. Our studies provide a robust and promising example of using in silico transcriptome-based screens for therapeutics to treat a common disease that is difficult to perform drug screens.

## RESULTS

### Generation of transcriptome-based cochlear signatures for acoustic trauma

**Fig. 1.**
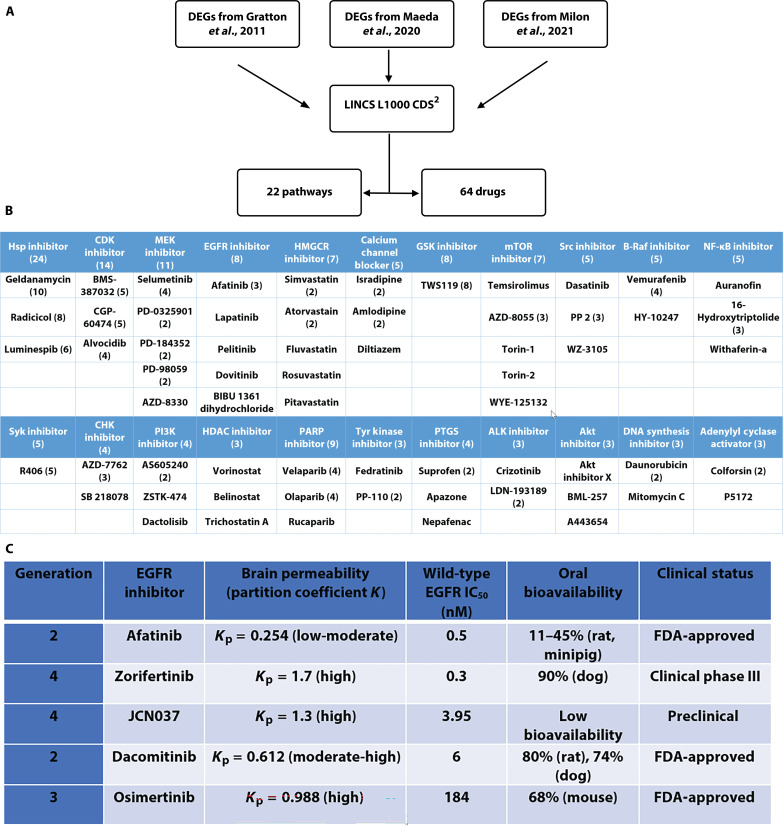
In silico screening identifies protective pathways and drugs against NIHL. (**A**) Workflow on in silico screens for pathways and drugs to protect against NIHL. (**B**) Top biological pathways and drug candidates identified against NIHL. HSP, heat shock protein; CDK, cyclin-dependent kinase; MEK, mitogen-activated protein kinase kinase; HMGCR, 3-hydroxy-3-methyl-glutaryl-coenzyme A reductase; GSK, glycogen synthase kinase; mTOR, mammalian target of rapamycin; NF-κB, nuclear factor κB; CHK, checkpoint kinase 1; PI3K, phosphatidylinositol 3-kinase; HDAC, histone deacetylase; PAPR, poly(ADP-ribose) polymerase; PTGS, prostaglandin-endoperoxide synthase; ALK, anaplastic lymphoma kinase. (**C**) EGFR inhibitors with proper safety and pharmacokinetics and pharmacodynamics profiles. IC_50_, median inhibitory concentration.

We used three sets of published in vivo transcriptome data (*40*–*42*) where differentially expressed genes (DEGs) in adult mouse cochleae with or without noise-exposure were obtained using microarray, NextGen RNA sequencing (RNA-seq) and single-cell RNA-seq analyses (Fig. 1A; see Materials and Methods). For the datasets of Gratton *et al*. (*40*) and Maeda *et al*., (*41*) DEGs were first identified using GEO2R and ranked based on *P* value and logFC: DEGs with a |logFC| > 2 and *P* < 0.05 were considered DEGs of interest to be included in further in silico analyses (fig. S1). For the dataset of Milon *et al*., (*42*) DEGs for OHCs, supporting cells, spiral ganglion neurons, and stria vascularis from the published lists were used without any further processing. In this dataset, a gene with absolute fold change (FC) >1.2 and a false discovery rate (FDR) *q* value < 0.05 was considered differentially expressed. Pathway analysis was performed on all DEGs of interest using ShinyGO Enrichment analysis, and Gene Ontology (GO) enrichment pathways were ranked on the basis of enrichment FDR values. LINCS L1000CDS^2^ was then compared for each GO enrichment pathway with at least three up-regulated and three down-regulated DEGs of interest, which resulted in a list of drug perturbations that could mimic or reverse the input gene expression in cancer cell lines. Drug perturbations were ranked on the basis of overlapping scores with the input gene list (file S1). Drugs with the highest overlapping scores were identified from each pathway, and drugs that targeted multiple significant pathways were considered more promising and advanced for in vivo studies. In total, 246 noveldrug perturbations were identified for the prevention and/or treatment of NIHL based on microarray and RNA-seq transcriptomic analysis.

### Ranking biological pathways and candidate drugs by their otoprotectant potential

After compiling the list of 246 novel drug perturbations, we determined each drug’s targets, predicted mechanisms of action, number of hits, and phase of FDA approval. L1000 Fireworks Display (L1000FWD) was used in conjunction with literature queries to determine which biological pathways are affected by each drug. Once the literature review was completed and the affected biological pathway was known for each drug, the pathways were ranked on the basis of the number of hits found in each pathway; pathways with the most hits are more strongly related to NIHL protection than pathways with fewer hits. This method of pathway ranking assumes that all the pathways involved will reverse the damage caused by noise rather than reversing possible protective pathways. Pathways with at least three hits were considered pathways of interest that require further study. Using this threshold, 22 biological pathways and 64 drugs targeting those pathways were identified (Fig. 1B).

Once the significant pathways related to NIHL were identified, our next step was to determine the drugs to be advanced for testing in animal models against noise exposure. We first focused on FDA-approved small molecule inhibitors in high-ranking pathways of mitogen-activated protein kinase kinase (MEK), EGFR, mammalian target of rapamycin (mTOR), Src, and histone deacetylase (HDAC), with excellent reported therapeutic properties and publications on other indications [i.e., pharmacokinetics and pharmacodynamics (PK/PD), maximum tolerated dose, blood-brain barrier (BBB) permeability (*43*), and preclinical and clinical status]. Among these inhibitors and pathways, many are known to be involved in NIHL (i.e., Braf, MEK, HDAC, and cyclin-dependent kinase (CDK)] (*22*, *27*, *44*, *45*), further validating our in silico screening strategies. For example, fluvastatin and lovastatin have been shown to protect against 110-dB noise exposure in CBA/CaJ mice (*46*). Suberoylanilide hydroxamic acid has been shown to attenuate NIHL (*44*); T-type calcium blockers have also been reported to show protection against NIHL (*47*).

We selected the EGFR inhibitors among our top otoprotectant candidates against NIHL from our noise transcriptomic LINCS analyses. Because EGFR is upstream of multiple pathways involved in NIHL, we conjectured that EGFR inhibitors may have better effects than those with individual pathways (i.e., Braf, MEK, HDAC, and CDK). We further searched additional EGFR inhibitors in the literature for high affinity to wild-type EGFR, high partition coefficient (*K*_p_) for brain permeability, high bioavailability, and already in clinical trials (Fig. 1C). Among the top five EGFR inhibitors identified, afatinib, a second generation EGFR inhibitor approved by FDA for cancer treatment, was the top-ranking drug in the enrichment analyses that were included in multiple pathways involved in the pathogenesis of NIHL. Zorifertinib (AZD3759) is a fourth-generation, BBB-penetrating EGFR inhibitor currently in cancer clinical trials. Molecular docking analyses further support that afatinib, zorifertinib, and other top EGFR inhibitors target the active kinase sites of EGFR (fig. S2).

### Exploring EGFR signaling in the adult mammalian cochlea

**Fig. 2.**
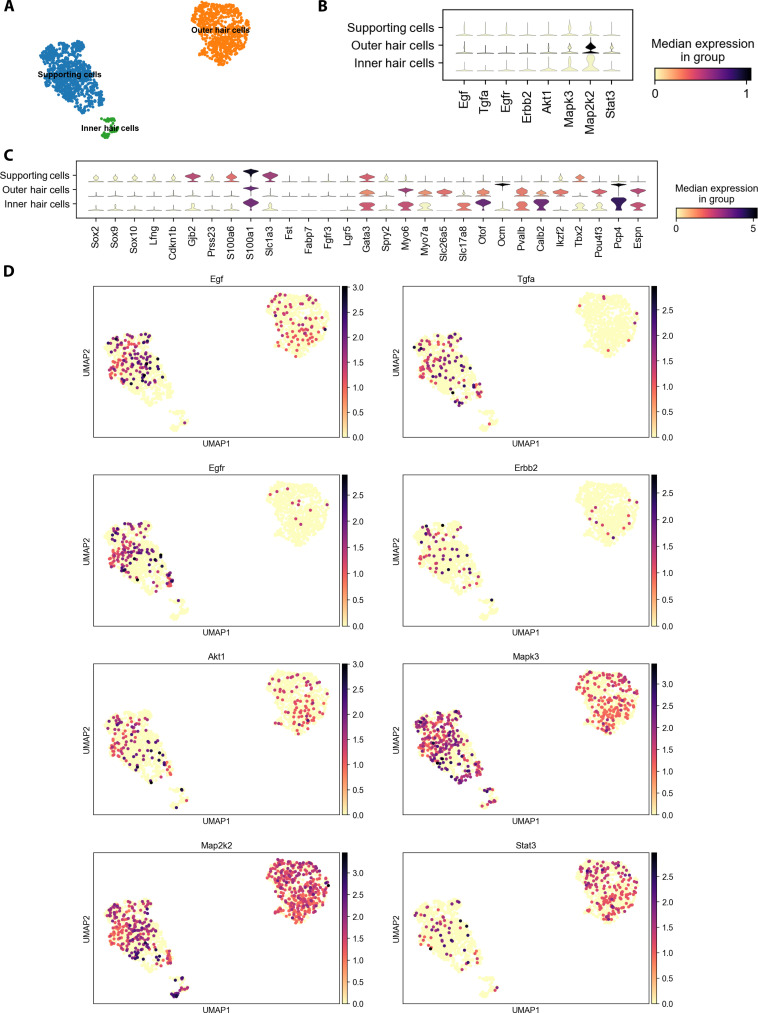
EGFR signaling in the adult mammalian cochlea. Single-cell RNA-seq reveals EGFR ligands, receptors, and downstream targets in hair and supporting cells. (**A**) Uniform Manifold Approximation and Projection plot with Leiden clustering showing supporting cell (blue), outer hair cell (orange), and inner hair cell (green) clusters in adult P28 C57BL6 mice. (**B**) Stacked violin plot showing hair and supporting cell markers in the clusters. (**C** and **D**) Expression levels and distribution of ligands (Egf and Tgfa), receptors (Egfr and Erbb2), and downstream targets (Akt1, Erk1/Mapk3, Map2k2, and Stat3) in the hair and supporting cell clusters. All single-cell RNA-seq analysis was done using Scanpy 1.9.3 (*116*). The code used is available at https://nbviewer.org/github/renevq/jupyter-notebooks/blob/main/Hang_data.ipynb. Similar results were obtained on the basis of our published dataset (*48*).

To provide evidence of expression of EGFR and downstream signaling pathways in the adult cochlear supporting cells and hair cells, we analyzed our published single-cell scRNA-seq datasets of P28 and P33 mouse cochlear supporting cells and hair cells (Fig. 2) (*48*, *49*). Representative EGF ligands (EGFL; Egf, Tgfa, Hbegf, Nrg1, Nrg4, and Btc) are all expressed in P28 and P33 supporting cells; EGFR family members (EGFR, ErbB2, ErbB3, and ErbB4) are expressed in P28 and P33 supporting cells and hair cells; and EGFR downstream signaling pathway genes (Akt/PI3k, Erk/Mapk, Stat3, and Plc) are expressed in P28 and P33 supporting cells and hair cells. These results are consistent with immunostaining results in adult cochleae (*50*, *51*). EGFR signaling expression is also known in stria vascularis, spiral ganglia, and stria ligament (*34*–*36*).

### Protective effects of EGFR inhibitors against hair cell excitotoxicity in zebrafish

Because there is no established mammalian cochlear explant assay that mimics noise injury, we adopted a zebrafish model that mimics hair cell damage due to excitotoxicity (*52*). Previous studies found that NIHL may be caused, in part, by glutamate excitotoxicity (*53*–*55*). Ionotropic glutamate receptor agonists have been used to mimic noise exposure in zebrafish larvae (*52*). We, therefore, tested the efficacy of the top five EGFR inhibitors (afatinib, zorifertinib, osimertinib, JCN037, and dacomitinib) to protect against kainic acid (KA)–induced excitotoxicity in this zebrafish lateral line neuromast model. This zebrafish excitotoxicity assay is by no means ideal for drug validation for NIHL but can serve as a high-throughput model for drug screening and validation before testing in mammalian models. If a drug is protective in both zebrafish and mouse models of NIHL, then it will provide evidence of conservation of mechanisms of action of the drug across species supporting human clinical use.

**Fig. 3.**
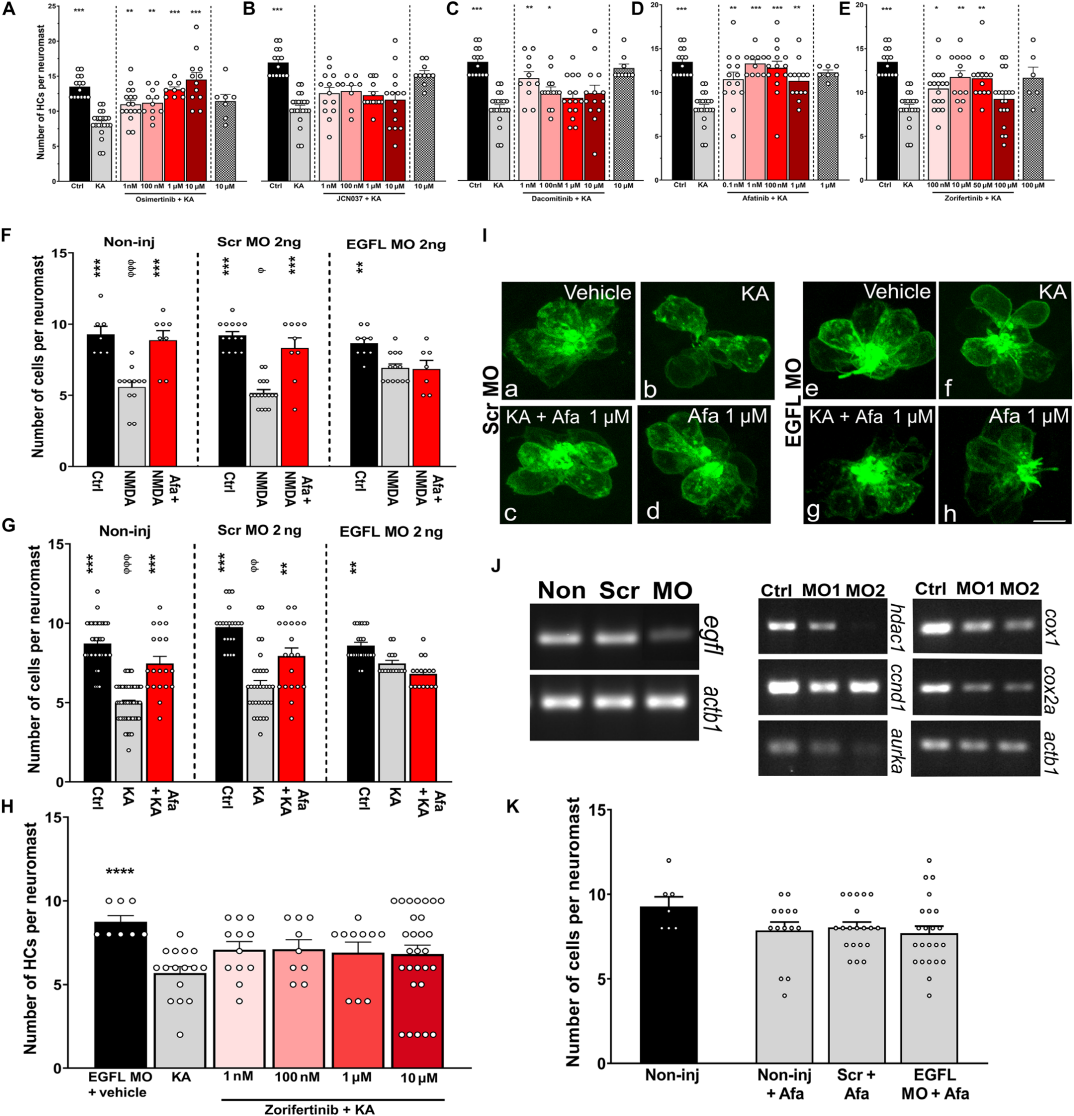
EGFR inhibitors protect against hair cell excitotoxicity in zebrafish. (**A** to **E**) Five- to 6-dfp zebrafish were incubated with 500 μM of KA for 1 hour followed by 2-hour incubation with different concentrations of EGFR inhibitors. (A) Osimertinib, (B) JCN037, (C) dacomitinib, (D) afatinib, and (E) zorifertinib. *P < 0.05, **P < 0.01, and ***P < 0.001 versus KA alone. (**F** to **K**) Zebrafish non-injected or injected with 2 ng of scrambled or EGFL-specific morpholinos. Animals (3 dpf) were preincubated with 500 μM of NMDA (F) or KA (G) for 1 hour followed by a 2-hour incubation with vehicle or afatinib (1 μM). Quantification of the HCs was performed in SO3, O1, and O2 neuromasts. **P < 0.01 and ***P < 0.001 versus ototoxin alone. ɸ*P* < 0.05, ɸɸ*P* < 0.01, and ɸɸɸ*P* < 0.001 versus the scrambled morpholino. (H) Zebrafish EGFL knockdowns (KDs) incubated with KA followed by different concentrations of zorifertinib. ****P < 0.01 versus KA alone. (I) Representative images of scrambled and EGFL morphants with the different treatments. Green (GFP) denotes the neuromast hair cells. Ctrl, control; KA + Afa 1 mM, KA and afatinib; Afa 1 mM, afatinib only. (J) Left gels: Confirmation of EGFL KD by reverse transcription polymerase chain reaction of scrambled and EGFL morphants. Right gels: EGFR downstream effectors after EGFL KD. Ctrl, control; MO1, EGFL MO 2 ng; MO2, EFGL MO 4 ng. hdac1, ccnd1, aurka, cox1, and cox2a. (K) Hair cell quantification in the different morphants under baseline conditions. Hair cell quantification is expressed as means ± SEM. *N* = 5 to 6 for each group. One-way ANOVA for (A) to (H) and (K).

Five to 6-day postfertilization (dpf) *Tg (brn3c:GFP)* zebrafish larvae were used for the assay. Fish were incubated with 500 mM of KA for 1 hour followed by various concentrations of the different EGFR inhibitors (Fig. 3, A to E). Except for JCN037 (Fig. 3B), all the EGFR inhibitors were protective at more than one concentration. To provide evidence that EGFR inhibitors act through the EGFR signaling pathway, we knocked down the EGFL in zebrafish. Not only was the EGFL transcript knocked down in the specific morphants (Fig. 3J, left) but also some of the EGFR downstream targets were affected. The downstream targets include hdac1, cyclin D1 (ccnd1), aurora kinase a (aurka), cytochrome c oxidase I (cox1), and cytochrome c oxidase II cox2a (Fig. 3J, right) (*56*–*58*). In addition, certain transcripts displayed a dose-dependent effect, specifically hdac1, aurka, and cox1. We tested protection against *N*-methyl-d-aspartate (NMDA) (Fig. 3F) and KA (Fig. 3, G to I) in the EGFL and scrambled morphants. As expected, the incubation of the EGFL knockdown (KD) animals with the EGFR inhibitors did not confer protection against excitotoxic damage (Fig. 3, F to I), suggesting that the protective effect is mediated via the EGFR pathway and that EGFR is the major pathway by which afatinib and zorifertinib protect against excitotoxicity. The use of two different EGFR inhibitors in combination with EGFL KD strongly points to the involvement of the EGFR signaling cascade during hair cell excitotoxicity. To be noted, there were no differences in the number of neuromast hair cells between non-injected, scrambled-injected, and EGFL-injected animals, confirming the lack of morpholino off-target effect (Fig. 3K).

### Otoprotection mediated by afatinib and zorifertinib against noise-induced hearing loss in mice

**Fig. 4.**
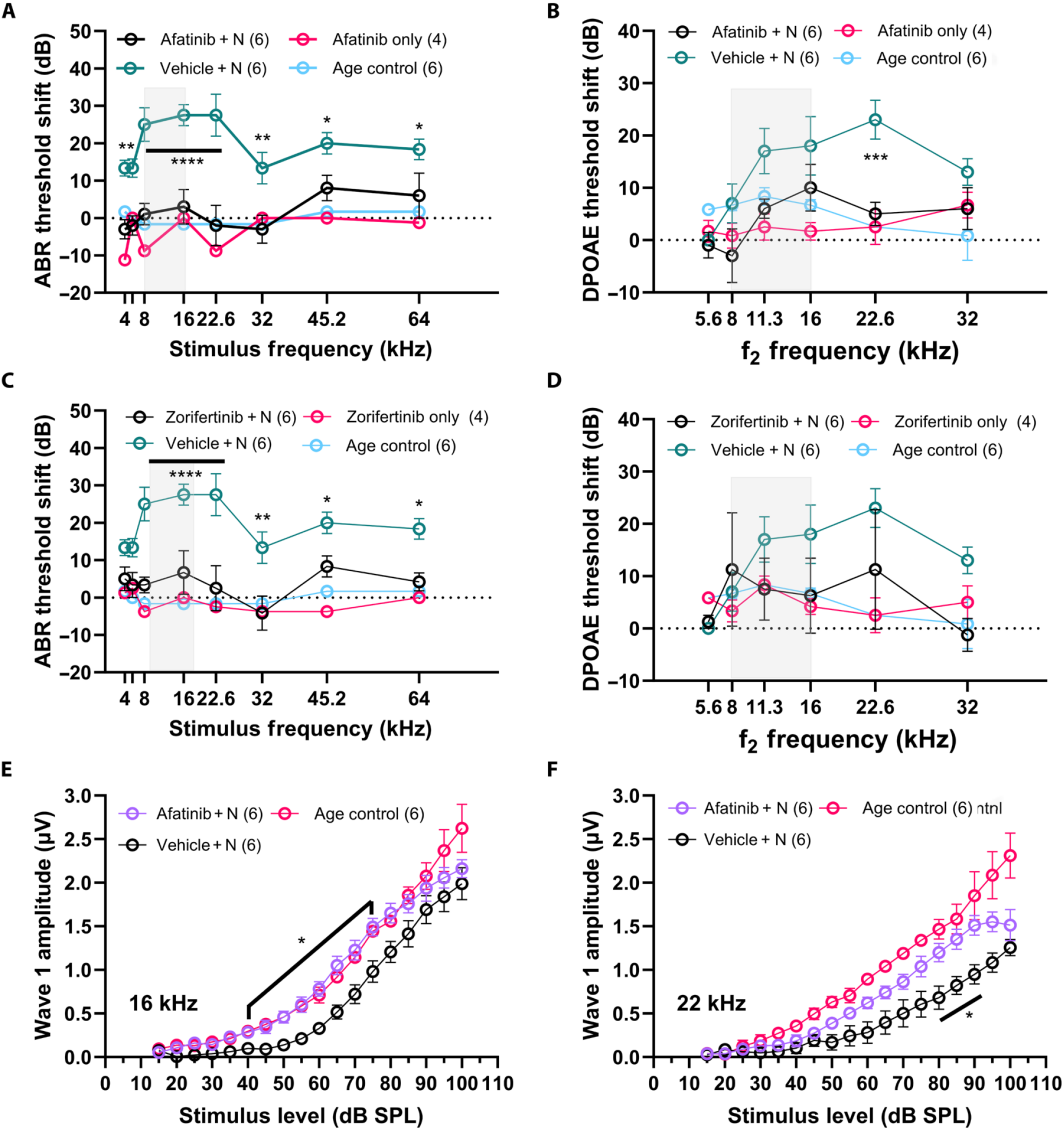
Afatinib and zorifertinib protect against noise-induced hearing loss in mice. Adult FVB mice (4 to 7 weeks old) were exposed to noise trauma (8- to 16-kHz noise band at 100-dB SPL for 2 hours) indicated by the shaded box in the figures. (**A**) ABR threshold shifts 2 weeks after the noise exposure. Animals treated with four doses of afatinib 20 mg/kg per day ip (magenta) showed significant differences in the threshold shifts at all tested frequencies when compared to those treated with the vehicle (teal) (4 kHz **P = 0.0037, 5.6 kHz **P = 0.0089, 8,16, and 22.6 kHz ****P < 0.0001; 32 kHz **P = 0.0044; 45.2 kHz *P = 0.0478; 64 kHz *P = 0.0395). (**B**) DPOAE thresholds (a function of the outer hair cells of the cochlea; defined as the lowest level of f2 that produced an emission amplitude of 0-dB SPL and was also 6 dB higher than the corresponding noise floor). A significant difference in threshold was observed at 22.6 kHz (***P = 0.0005). (**C**) ABR threshold shifts 2 weeks after noise exposure. Animals treated with 5 doses of zorifertinib 15 mg/kg oral gavage showed significant protection at all tested frequencies above 16 kHz (8,16, and 22.6 kHz ****P < 0.0001; 32 kHz **P = 0.0011; 45.2 kHz *P = 0.0447; 64 kHz *P = 0.0112). (**D**) DPOAE threshold difference did not reach statistical significance. (**E** and **F**) ABR wave 1 amplitudes for 16- and 22-kHz stimuli respectively showed significantly larger amplitudes at suprathreshold levels in the afatinib-treated animals compared to the vehicle-treated (*P = 0.0085 to 0.0498 for 16 kHz and *P = 0.0233 to 0.0352 at 80- to 90-dB SPL for 22 kHz). Two-way ANOVA, Holm-Šidák post hoc test. Data are presented as means ± SEM, *n* = 4 to 6 per group. “+ N” indicates with noise exposure.

The otoprotective effects of afatinib and zorifertinib were further evaluated in a mouse model of acute NIHL. We used a previously established permanent threshold shift (PTS) FVB mouse model (*27*) to test the otoprotective effect. After baseline auditory function evaluation using auditory brainstem response (ABR) and distortion product otoacoustic emission (DPOAE) measurements, mice were exposed to 100-dB sound pressure level (SPL) 8- to 16-kHz octave band noise for 2 hours. Experimental animals were given either afatinib [20 mg/kg ip (intraperitoneally) delivery] or zorifertinib (15 mg/kg oral delivery) 1 day before the noise exposure and then continued for the following 4 days. Treatment with afatinib or zorifertinib significantly attenuated PTSs 14 days after noise exposure compared to the vehicle-treated group (Fig. 4, A to D). Drug-only–treated animals did not show any significant difference in the threshold shifts. ABR threshold shifts were significantly attenuated at 16-, 22.6-, 32-, 45.2-, and 64-kHz frequencies for both drug treatment groups (Fig. 4, A and C). DPOAE threshold shifts were most prominently protected in afatinib-treated animals at 22.6 kHz (Fig. 4B). Although zorifertinib-treated animals also showed lower DPOAE threshold shifts compared to the vehicle-treated group, it did not reach statistical significance (Fig. 4D). In addition, significant differences were observed at multiple stimulus levels in ABR wave 1 amplitude input-output function at both 16- and 22.6-kHz frequencies in the afatinib-treated group (Fig. 4, E and F).

### Mitigation of cochlear synaptopathy in mice by afatinib and zorifertinib

**Fig. 5.**
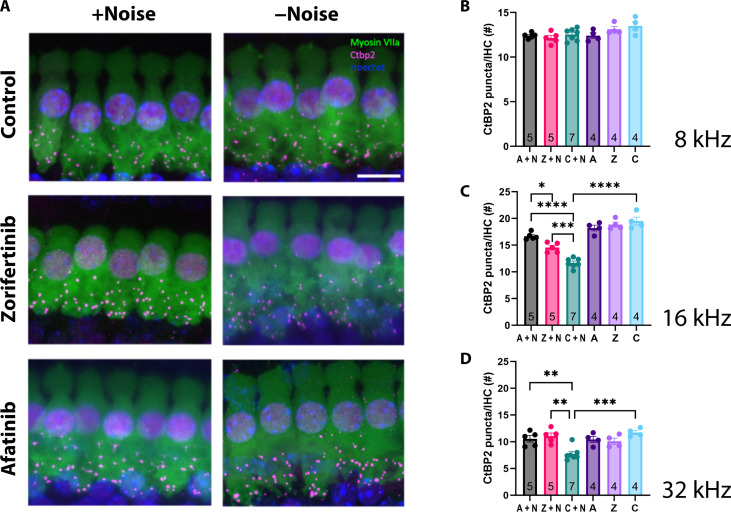
Afatinib and zorifertinib protect against noise-induced cochlear synaptopathy in mice. (**A**) Representative maximum intensity projections of the 16- to 22-kHz region of the cochlea following drug treatment and with (left) or without noise (right). Hair cells were labeled using Myosin VIIa, presynaptic puncta were labeled using CtBP2, and nuclei were counterstained with Hoechst. (**B** to **D**) Zorifertinib and afatinib protect against noise-induced cochlear synaptopathy, resulting in significantly less CtBP2 puncta loss with drug + noise than control + noise. Individual data points are the average of the left and right cochlea from each mouse. A, afatinib only; Z, zorifertinib only; C, control (age-matched); A + N, noise-exposed group treated with afatinib; Z + N, noise-exposed group treated with zorifertinib; C + N, noise-exposed group treated with vehicle for the drugs. Data are presented as means ± SEM. The sample size is indicated inside the vertical bars for each group. One-way ANOVA with Bonferroni’s post hoc test. 16 kHz: A + N versus C + N ****P < 0.0001, Z + N versus C + N ***P = 0.0008, C + N versus C ****P < 0.0001, A + N vs Z + N *P = 0.0353; 32 kHz: A + N versus C + N **P = 0.0071, Z + N versus C + N **P = 0.0012, C + N versus C ***P = 0.0003. Scale bar, 10 μm.

In the acute NIHL mouse model, moderately loud noise exposure did not produce significant hair cell loss (fig. S3) (i.e., 100-dB SPL 8- to 16-kHz exposure for 2 hours in FVB mice) which is consistent with previous reports (*27*) but is associated with permanent loss of cochlear afferent synapses on the IHCs (*27*, *59*, *60*). Therefore, we examined and quantified synaptic ribbons in the IHCs in three tonotopic regions of the cochlea, 8- to 10-kHz, 16- to 22-kHz, and 32- to 40-kHz regions (Fig. 5). Without noise, we found no significant difference in the number of CtBP2 presynaptic puncta among the zorifertinib, afatinib, and age-matched control groups in the three tonotopic regions (Fig. 5, B to D). However, after noise exposure, there were significantly more CtBP2 presynaptic puncta in the zorifertinib and afatinib-treated groups compared to the control group exposed to noise and treated with the vehicle in 16- and 32-kHz regions (Fig. 5, C and D), suggesting that zorifertinib and afatinib protect against noise-induced cochlear synaptopathy. No significant difference was observed in the 8-kHz region. Although the synaptic loss was significantly lower in the drug-treated groups, it was not fully restored to the level seen in age-matched control for the 16-kHz region (Fig. 5C).

### Protective effect of conditional knockout of EGFR against NIHL

**Fig. 6.**
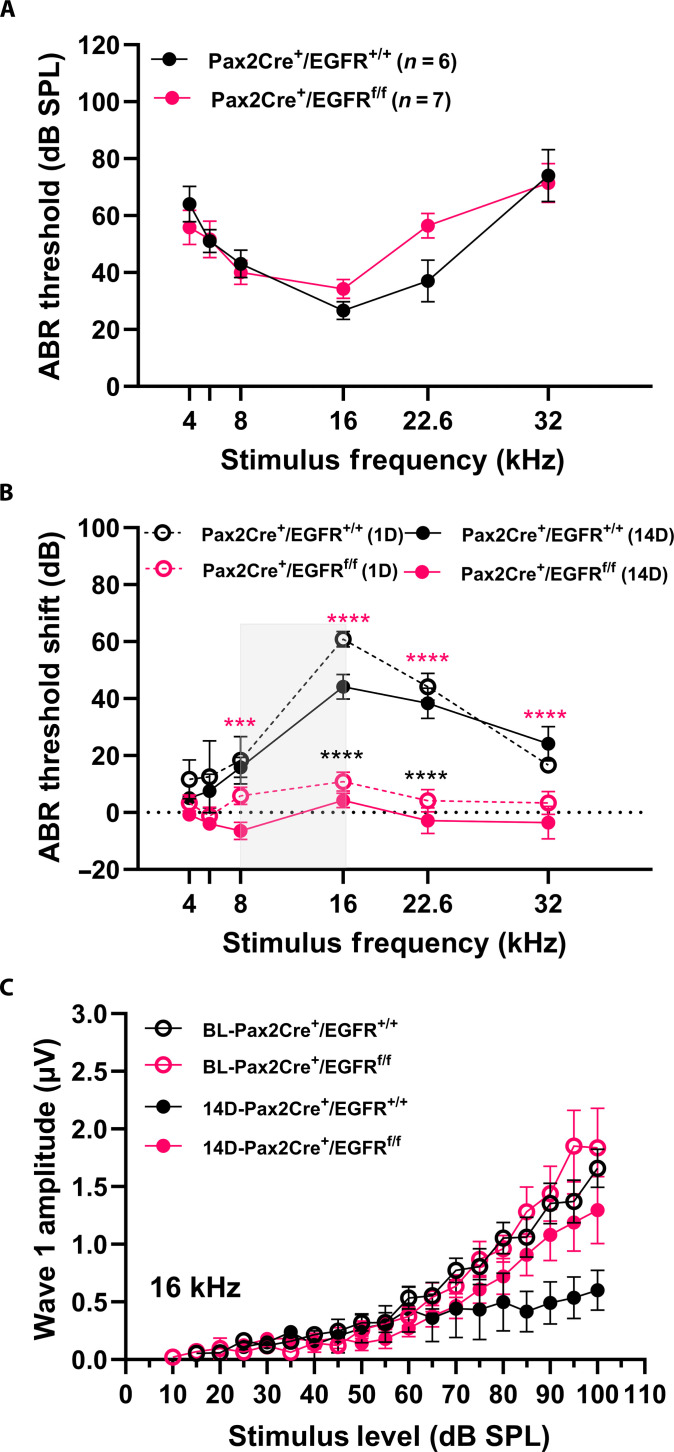
Conditional knockout of EGFR shows protection against NIHL. (**A**) Baseline (BL) (pre-noise exposure) ABR thresholds of Pax2Cre; EGFR^flox/flox^ mice and Pax2Cre; EGFR^+/+^ control littermates measured at 4 to 6 weeks of age. The mice were on a mixed background of CD1, CBA/CaJ, and C57BL/6 strains. No significant differences were detected between cKO and WT; *n* = 6 to 7. (**B**) Pax2Cre; EGFR^flox/flox^ mice (*n* = 6) exhibited significantly smaller ABR threshold shifts [both 1 day (1D) for TTS and 14 days (14D) for PTS) than wild-type (WT) littermate controls (Pax2Cre; EGFR^+/+^; *n* = 6) C) ABR wave 1 amplitudes for 16 kHz stimuli respectively showed larger amplitudes at suprathreshold levels in the cKO compared to the WT but did not reach statistical significance. Two-way ANOVA, Holm-Šidák post hoc test; ****P < 0.0001 and ***P = 0.0007; black (1D) and magenta (14D). Data are presented as means ± SEM.

To further validate the protective effect of inhibition of EGFR signaling against NIHL, we generated a conditional knockout (cKO) mouse strain of EGFR by crossing EGFR floxed mice with Pax2-Cre mice. The Pax2-Cre; EGFR cKO mice showed normal hearing thresholds compared to their wild-type littermates (Fig. 6A). No apparent differences in hair cell counts or morphology were observed between the wild-type and homozygous mutants (fig. S4, A and B). To test whether the Pax2-Cre; EGFR cKO mice were protected against NIHL, we exposed 4- to 6-week-old mice to 2 hours of 100-dB SPL 8- to 16-kHz octave band noise, like the noise exposure in the afatinib and zorifertinib-treated groups. cKO mice displayed significantly smaller ABR threshold shifts at 8-, 16-, 22.6-, and 32-kHz frequencies on both 1 and 14 days after the noise exposure compared to the wild-type littermate controls (Fig. 6B). Wave 1 amplitudes of ABRs at 16 kHz were larger but did not reach significance in the cKO mice (Fig. 6C). Cochlear morphological analysis suggested protection against synaptic loss as evidenced by a greater number of presynaptic CtBP2 puncta in the homozygous cKO mice compared to the wild type (fig. S4, C and D). These results are similar to the protection against NIHL offered by pharmacological inhibition of EGFR signaling (afatinib and zorifertinib; Fig. 4) in the zebrafish.

### Activation of EGFR signaling in the mouse cochlea following noise exposure and its attenuation by zorifertinib

**Fig. 7.**
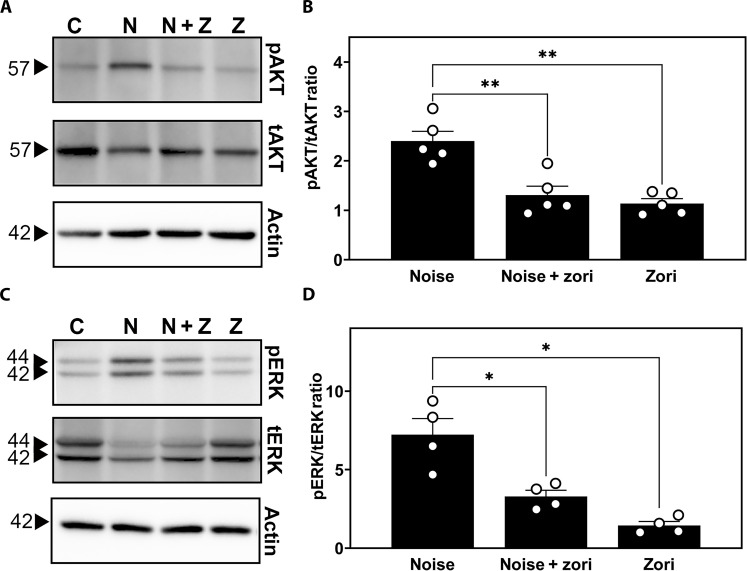
EGFR signaling is activated in the mouse cochlea following noise exposure and attenuated by zorifertinib. (**A** to **D**) Western blotting was performed on organ of Corti lysates from mice 30 min after exposure to noise trauma (8- to 16-kHz noise band at 100-dB SPL for 2 hours) and pretreated with either the drug (zorifertinib 15 mg/kg, oral) or the vehicle (normal saline or methyl cellulose) 1 day before and 1 hour during the noise trauma to detect the phosphorylation status (p, phosphorylated versus t, total) of two downstream effectors of EGFR (AKT and ERK). Zorifertinib pretreatment significantly decreased the noise-induced increase of AKT and ERK phosphorylation. [(A) and (C)] The sizes of the bands are labeled in kilodaltons. C, untreated and unexposed control; V + N, vehicle + noise; Z + N, zorifertinib + noise; Z, zorifertinib only. Actin: A loading control. [(B) and (D)] Ratios of phosphorylated versus total proteins normalized to actin loading controls. Data are normalized to untreated/exposed control mice as a ratio of 1 and presented as means ± SD. *N* = 4 to 5 biological replicates and each dot represents one mouse in each group. ANOVA, Tukey’s post hoc. *P ≤ 0.05 and **P ≤ 0.01.

To determine whether EGFR signaling is functional in the adult cochlea, we treated wild-type FVB mice with zorifertinib (oral gavage at 15 mg/kg per day) a day before and at 1 hour during noise trauma. We exposed the mice to noise trauma (100-dB SPL 8 to 16 kHz for 2 hours) and performed immunoblot analysis for downstream signaling targets of EGFR on cochlear lysates collected 30 min after the noise trauma. We found that extracellular signal–regulated kinase (ERK) and AKT phosphorylation were induced 30 min after noise exposure but significantly reduced by the drug treatments (Fig. 7, A to D). Our results corroborate with previous publications on noise-induced activation of ERK and AKT pathways in similar postexposure time points (*61*–*64*). Specifically, ERK phosphorylation in supporting cells is activated in cisplatin-treated but reduced in otoprotective drug-treated mice (*22*). Together, these results strongly indicate that EGFR signaling is functional and responsive to noise exposure. EGFR inhibitors are effective in mitigating the noise-induced activation of AKT/ERK signaling pathways in the adult mouse cochlea in vivo.

**Fig. 8.**
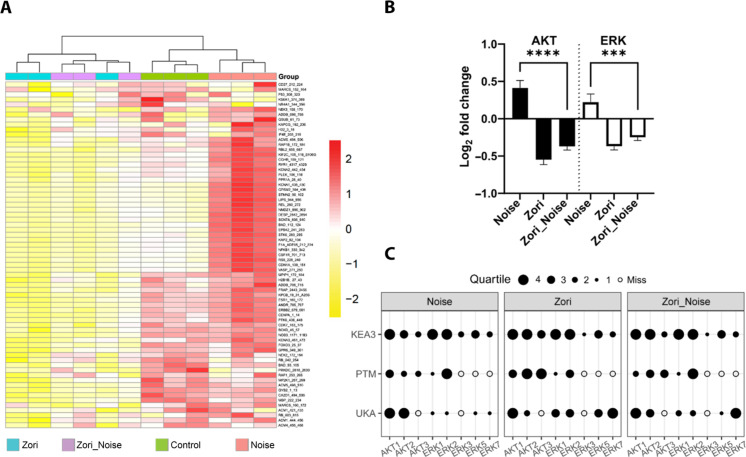
Zorifertinib suppresses noise-activated EGFR signaling pathways in mouse cochleae. (**A**) Heatmap of phosphorylation activity at the reporter peptides on the kinome panel (STK PamChip; *n* = 3 chips run in technical triplicate). Cochlear sensory epithelial lysates were collected 30-min after noise exposure from control, noise-exposed, zorifertinib-treated (Zori), and zorifertinib-treated plus noise-exposed (Zori_Noise) adult FVB mice. Each row represents a peptide. The protein kinase activity measured as phosphorylation of reported peptides on the chip is scaled with red being higher activity and yellow indicating lower activity. (**B**) Log_2_ fold activity changes of reporter peptides “mapped” as putative targets of AKT and/or ERK kinase families (24 and 25 peptides, respectively) expressed as means ± SD. An average of triplicates was used for each peptide per condition. N/C, noise versus control; Z/C, zorifertinib versus control; N + Z/C, noise + zorifertinib versus control. Bars, SEM; ****P < 0.0001 and ***P < 0.001, two-way ANOVA. (**C**) Identification of specific protein kinases using complementary software packages (details in the Supplementary Materials). Selected AKT and ERK family members were identified as candidate “hits” using deconvolution strategies that specify protein kinases that are contributing to the phosphorylation signal seen across all reporter peptides on the STK chip. Upstream kinase analysis (UKA), posttranslational modification signature enrichment analysis (PTM-SEA), and kinase enrichment analysis 3 (KEA3) were used to identify specific kinases within the AKT and ERK families using online phosphosite mapping databases. Data were normalized and grouped as quartiles (1 to 4 black dots) for visualization using our bespoke Creedenzymatic R package.

To further elucidate the mechanisms of action by zorifertinib against noise trauma in the adult mouse cochlea, we used a novel kinome array to compare kinase signaling pathways in the cochlear lysates in mice treated with zorifertinib and/or noise trauma (see Materials and Methods). Specifically, a serine/threonine kinase (STK) PamChip containing 144 immobilized peptides which were used as a readout of kinase activity (fig. S6). Adult FVB mice were treated with zorifertinib (oral gavage at 15 mg/kg per day 1 day prior and 1 hour during noise trauma) and exposed to noise (100-dB SPL 8 to 16 kHz for 2 hours), and cochlear lysates were collected 30 min after noise exposure, in conditions identical to Western blot analysis. We found that technical triplicates of each condition were highly reproducible (Fig. 8A and figs. S7 to S16), zorifertinib suppressed multiple signaling pathways, and signaling downstream of EFGR was higher in noise-exposed conditions but treatment with zorifertinib suppressed noise-induced activation of AKT/ERK signaling activities 30 min after noise exposure (Fig. 8B). Using various deconvolution strategies (upstream kinase analysis [UKA], posttranslational modification signature enrichment analysis [PTM-SEA], and kinase enrichment analysis 3 [KEA3]; see Materials and Methods), we identified selected AKT and ERK family members as candidate “hits” that are contributing to the phosphorylation signal seen across all reporter peptides on the STK chip (Fig. 8C). These results are consistent with our Western results (Fig. 7, A to D) and previous publications under similar conditions, thus confirming that inhibition of the EGFR signaling pathway is a mechanism of action by zorifertinib against NIHL in the adult mouse cochlea.

### Pharmacokinetics of orally delivered zorifertinib in mouse inner ear perilymph fluids

**Fig. 9.**
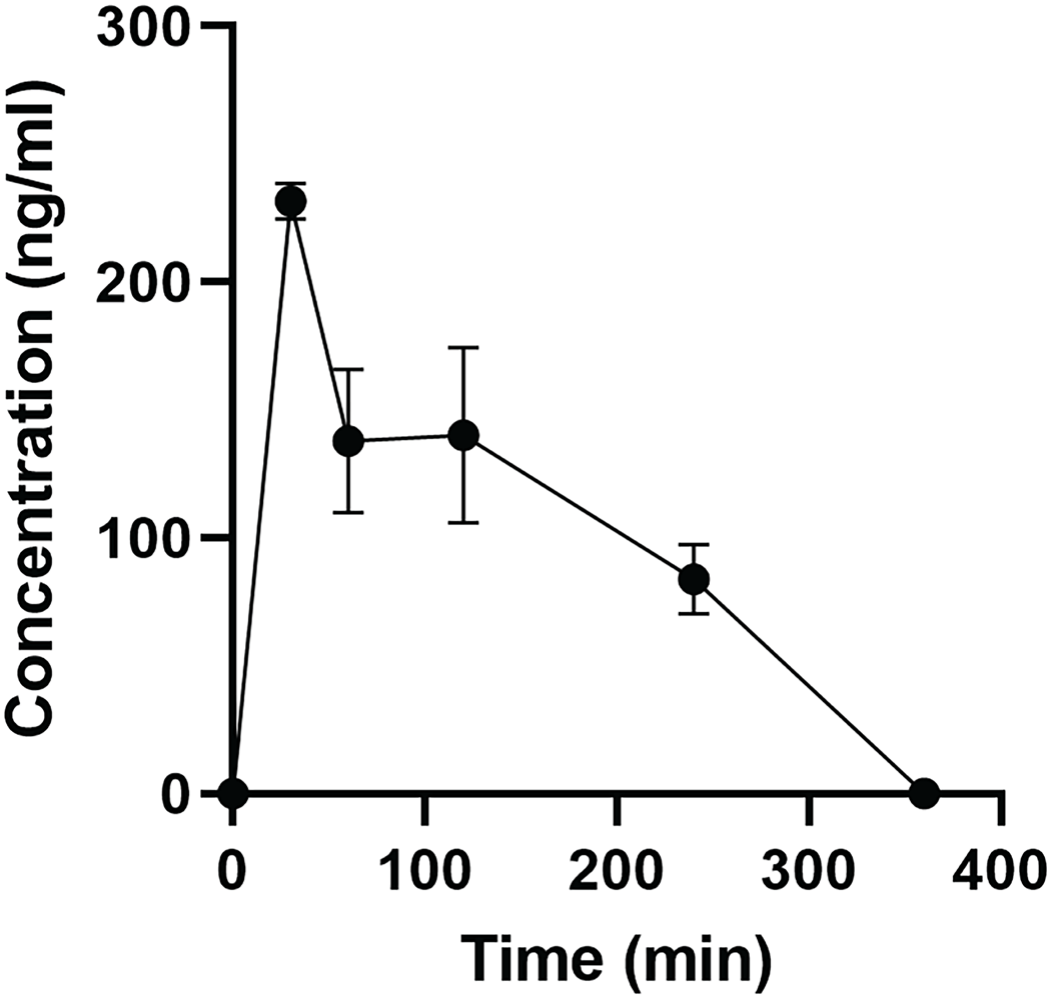
Pharmacokinetics of orally delivered zorifertinib in mouse inner ear perilymph fluids. Measured concentrations of zorifertinib in mouse perilymph 0 to 6 hours after oral gavage (15 mg/kg; *n* = 3 per time point). Error bars indicate SEM. PK properties are calculated: *T*_max_ = 0.5 hours (time of maximum perilymph concentration), *T*_1/2_ = 2.1 hours (half-life), *C*_max_ = 99.9 ± 62.6 ng/ml (maximum concentration), AUC_0-*t*_ = 189.9 ± 48.3 ng/ml*hour (area under the curve), *V*_Z_/*F* = 0.19 (volume of distribution), Cl/*F* = 0.06 (mg/kg)/(ng/ml)/hour (clearance).

To validate that orally delivered zorifertinib crosses the blood-labyrinth barrier (BLB) of the inner ear, we performed perilymph collection of adult FVB mice at various time points after oral gavage of zorifertinib (15 mg/kg). To accurately measure the perilymph concentration of zorifertinib, we performed liquid chromatography–tandem mass spectrometry (LC-MS/MS) measurement of zorifertinib in ~1 μl of perilymph fluid collected from each mouse using crizotinib as an internal standard (IS); the calibration curve showed linearity within the measured range, with a lower limit of quantification of 5 ng/ml (fig. S17). The time course of zorifertinib concentrations in perilymph is shown in Fig. 9. The *C*_max_ was 100 ng/ml and *t*_max_ was 30 min after oral gavage, while *t*_1/2_ was ~130 min and zorifertinib was cleared from perilymph in 6 hours. These PK results in the inner ear fluids are consistent with the protection of zorifertinib we observed in mice with noise exposure (Fig. 4, C and D).

### Synergistic effects of zorifertinib and AZD5438 in zebrafish

**Fig. 10.**
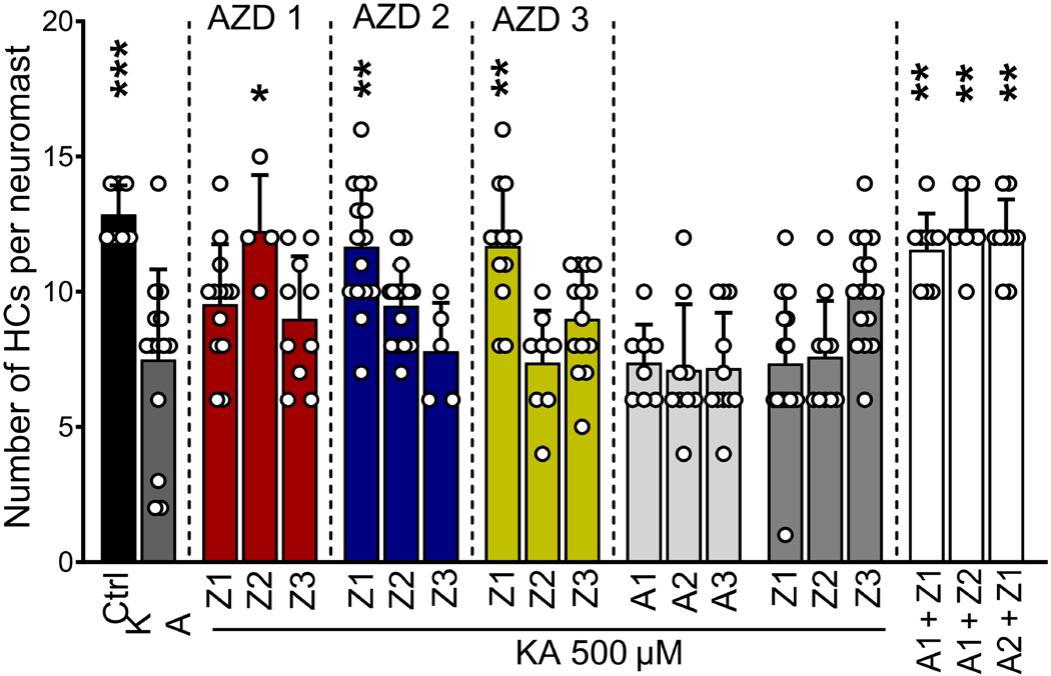
Synergistic otoprotection between zorifertinib and AZD5438 against excitotoxicity in zebrafish neuromasts. Five-dpf zebrafish were preincubated with KA (500 μM) or regular fish water for 1 hour and then incubated for 2 hours with a combination of AZD5438 (CDK2 inhibitor) and zorifertinib (EGFR inhibitor) or with the individual compounds. Results are expressed as the number of hair cells per neuromast ± SD. *P < 0.05, **P < 0.01, and ***P < 0.001 versus KA alone (one-way ANOVA). A1, AZD5438 50 nM; A2, AZD5438 1 nM; A3, AZD5438 10 pM; Z1, zorifertinib 50 nM; Z2, zorifertinib 1 nM; Z3, zorifertinib 10 pM.

Given the multiple pathways involved in the pathophysiology of NIHL, we hypothesized that the EGFR inhibitor could synergize with an inhibitor of the CDK2 pathway and thus increase the levels of protection against NIHL. For this purpose, we used the zebrafish model for glutamate excitotoxicity to test the protective effect of zorifertinib in the presence of AZD5438, a CDK2 inhibitor that we have characterized before as an otoprotective compound against NIHL (*22*). Zebrafish larvae were preincubated with 500 μM KA or control fish water for 1 hour followed by incubation with a combination of varying doses of zorifertinib and AZD5438. Dose combinations of zorifertinib (1 nM) + AZD5438 (50 nM), zorifertinib (50 nM) + AZD5438 (1 nM), and zorifertinib (50 nM) + AZD5438 (50 nM) showed synergistic protection compared to treatment with the individual drugs (Fig. 10). These results demonstrate that zorifertinib and the CDK2 inhibitor, AZD5438, act in synergy against NIHL via inhibiting both EGFR and CDK2 signaling pathways.

## DISCUSSION

Here, we used a novel strategy for the discovery of otoprotective compounds using cochlear transcriptomes associated with noise exposure and comparing those to a database of drug-induced transcriptomic changes in cell lines. We identified 22 biological pathways and 64 drugs that have the potential as otoprotectants to reverse the transcriptomic changes associated with noise exposure. We successfully validated in animal models EGFR inhibitors as promising otoprotective drugs for the treatment of NIHL.

### In silico screens for therapeutic drugs and biological pathways for hearing loss

Accelerating drug discovery for preventable conditions like NIHL is of paramount importance than ever before. Ever since the CMap (*30*) was first introduced, this method has been used in pharmacological research to establish connections between disease states and drugs (*65*–*67*). This method has also been used by other scientists in their quest to discover potential new treatments for various types of cancer, along with rare conditions like Hirschsprung disorder and most recently for COVID-19 (*68*–*73*).

The overarching hypothesis of our study was that compounds able to revert the expression of genes associated with noise damage may be able to revert or prevent NIHL. EGFR signaling is novel in NIHL and additional downstream targets can provide additional NIHL drug candidates and biomarkers for NIHL pharmacokinetic analysis. Combinatory treatments of drugs targeting EGFR and CDK2 signaling pathways are more effective in otoprotection than individual drug treatment in the zebrafish model here, a result that corroborates with our recent studies using a combination of Braf and CDK2 inhibitors in preventing NIHL and cisplatin-indued hearing loss in mice (*22*).

### Repurposing FDA-approved drugs for preventing NIHL

Many benchmark candidates (sodium thiosulfate, ebselen, *N-*acetylcysteine, and d-methionine originally chosen as antioxidants against neurodegeneration) have shown some promise in preclinical and clinical trials against NIHL (*74*); however, to date, no drugs have been approved by the FDA for clinical use to prevent any forms of NIHL. Repurposing FDA-approved drugs offers many advantages over developing new chemical entities such as cost- and time-effectiveness and known safety/pharmacokinetics profiles (*22*, *25*, *27*, *75*, *76*). Repurposing drugs can also be further expedited for orphan diseases (e.g., pediatric cisplatin-induced hearing loss).

### EGFR inhibitors as candidate drugs for clinical trials on hearing loss

We selected EGFR inhibitors as our top otoprotectant candidates against NIHL from our noise transcriptomic LINCS analysis. In the enrichment analyses, afatinib was the top-ranking drug that was included in multiple pathways suggested to be involved in the pathogenesis of NIHL. Afatinib is an FDA-approved second-generation, irreversible, dual EGFR and Her2 inhibitor, with an IC_50_ (median inhibitory concentration) of 0.5 and 14 nM, respectively, and with excellent kinase selectivity (*77*). It is FDA-approved as the first-line treatment for patients with advanced/metastatic non–small cell lung carcinoma (NSCLC) carrying EGFR mutations (*78*). Additional generations of EGFR inhibitors have been recently characterized with even better specificity, potency, PK/PD properties, bioavailability, and BBB permeability (Fig. 1C). We selected and tested the otoprotective effect of zorifertinib, a fourth-generation inhibitor that has excellent BBB permeability and bioavailability (*79*, *80*). Zorifertinib is currently in clinical development for the treatment of NSCLC with CNS metastases (*81*). Both afatinib and zorifertinib plus two other EGFR inhibitors (osimertinib and dacomitinib) showed individually excellent protection, in multidoses, against glutamate excitotoxicity that mimics noise damage in the zebrafish lateral line neuromast model (Fig. 3). Furthermore, we have shown that combinatorial treatment with zorifertinib and a specific, potent CDK2 inhibitor AZD5438, exhibits synergistic otoprotection in the zebrafish model (Fig. 10). We tested protection against NIHL using a PTS noise exposure paradigm in a mouse model. Pretreatment with afatinib or zorifertinib provided 20 to 30 dB of otoprotection at all tested frequencies (Fig. 4). Exposure to octave band noise in mice reportedly produces maximum damage in cochlear regions that are one-half to two octaves higher than the exposure stimulus (*82*). In this study, significant otoprotection was observed even in the 64-kHz regions. To our knowledge, this is one of the first studies that has demonstrated otoprotection in the high-frequency cochlear region. Our mass spectrometry results provide direct evidence that zorifertinib crosses the BLB (Fig. 9). Zorifertinib displayed excellent pharmacokinetic properties that are desirable for BBB/BLB-penetrating drugs (*83*). Our Pax2-Cre; EGFR cKO mouse (Fig. 6) and EGF KD zebrafish (Fig. 3) studies exhibited otoprotection against NIHL, similar to afatinib and zorifertinib; EGF KD plus afatinib in zebrafish did not show additional protection to that by either afatinib or EGF KD alone. Together, these results confirmed that EGFR plays a key role in NIHL and mediates EGFR inhibitors’ otoprotection in vivo. Our results further demonstrate that EGFR inhibitors are an excellent class of drug candidates for NIHL and thus ready for advancing to clinical trials on hearing loss. Additional newer EGFR inhibitors with better specificity and PK/PD characters than afatinib and zorifertinib may therefore have potential as otoprotectants against NIHL and other forms of hearing loss.

### Mechanisms of action by afatinib and zorifertinib for otoprotection against NIHL

It has been well documented that afatinib and zorifertinib are potent EGFR inhibitors; however, it is unclear whether they act in similar mechanisms in cochleae against NIHL. To address this, we first confirmed that phosphorylation of AKT and ERK (two main downstream targets of EGFR) are activated by noise exposure but mitigated by zorifertinib treatment. We further performed an unbiased kinome analysis of mouse cochlear lysates using a novel kinase peptide array (*84*, *85*). This experiment revealed multiple kinase signaling pathways that are altered by noise exposure and ameliorated by zorifertinib in mouse models of NIHL (Fig. 8, A to C, and figs. S6 to S16), consistent with our Western results (Fig. 7, A to D). Our Western and kinome results are also consistent with previous publications on noise-induced activation of several signaling pathways [AKT and ERK; (*61*–*64*)]. Moreover, our kinome results are consistent with a recent study using proteomics under similar noise exposure in mice (*86*). Compared to previous studies, our kinome analysis uncovers a wider range of signaling pathways including more than 340 kinase targets/reporters, most of which are active in cochleae in response to noise exposure. It will be interesting to further examine lower doses of zorifertinib as well as various levels and durations of noise exposure to accurately profile the specific kinase pathways that are at work for NIHL.

Our results further support that inhibition of EGFR has effects at least similar to (if not more potent than) inhibition of individual downstream targets and that combinations of EGFR and additional inhibitors of downstream or synergistic pathways (i.e., AZD5438 for CDK2) are more effective than individual inhibitors in protecting against NIHL. Moreover, it remains unclear which cell types mediate EGFR effects during noise exposure. Given that EGFLs, EGFRs, and downstream targets are expressed in both supporting cells, hair cells, and spiral ganglia (Fig. 2), it remains to be further studied how EGFR signaling mediates NIHL at cellular levels. It would be interesting to compare changes in kinome signaling pathways under other ototoxic insults (cisplatin, antibiotics, and aging) so that both common and specific cochlear mechanisms of action can be validated, and corresponding therapeutic strategies can be developed.

An increasing number of recent studies have unexpectedly revealed that signaling pathways normally controlling cell proliferation in cancers are also involved in stress-induced cell death in postmitotic, wild-type cells (*22*, *25*, *27*, *75*, *76*). For example, CDK2 and Braf inhibition protected against ototoxic insults in postmitotic cochlear cells as we have demonstrated here on EGFR inhibition (*22*). These studies together suggest that pharmaceutical inhibition of cell proliferation pathways (i.e., EGFR, CDK, and Braf) may have similar protective effects against stresses in nervous and other postmitotic systems.

### Limitations

Our in silico screens are based on transcriptomic changes of mostly drug-treated cancer cell lines in CMap that heavily reflect drug effects on cell proliferation, while additional pathways are likely involved in stress responses that remain to be further identified. In addition, our screens assumed that pathways and drug hits are directly involved in reversing the damage caused by noise rather than reversing possible protective pathways. From the list of 22 biological pathways and 64 drug candidates in our in silico screens, many have been tested as otoprotective in various in vitro or in vivo models; however, we have only tested one top pathway (EGFR) and its inhibitors. It remains to be further tested if other pathways and drugs work under similar NIHL conditions. Moreover, the doses at which zorifertinib and afatinib were tested could induce multiple nonspecific pathways as evidenced in our kinome panel results; it is desirable to test lower doses of drugs for specific pathways involved. It is important to further study EGFR signaling changes upon noise exposure and drug treatments at single-cell levels to further advance Western and kinome results. Hair bundle morphology and postsynaptic density remain further examined upon noise and EGFR inhibitor treatment. Given the importance of efferent in noise protection in mice, EGFR inhibitors’ potential roles in efferent modulation can be further tested in the future. Using a second Cre line that targets SGN (type Ia) would be informative. ATF3/4 and STAT3/IRF7 could be involved in EGFR inhibitors’ effects, an interesting hypothesis to be tested in the future. Our kinome results using afatinib could also collaborate with those of zorifertinib. Compared to mice and other rodents used for preclinical studies, humans seem to be less susceptible to NIHL, especially those that only produce temporary threshold shifts (TTSs). In a recent report that compared TTSs in humans and mice, a difference of 15 dB in noise vulnerability was noted (*87*). A direct translation of noise exposure levels in animals to humans is also challenging due to the differences in the noise exposure that humans experience in the real world. These factors need to be considered when translating the results of animal studies into clinical trials. Last, we only tested one noise exposure condition and one drug regimen in mice; it is important to test additional levels, durations of noise, and drug regimens to further elucidate the full potential of EGFR inhibitors in protecting against various forms of NIHL.

## MATERIALS AND METHODS

### Ethics statement

Care and use of animals follow the guidelines in the National Institutes of Health Guide for the Care and Use of Laboratory Animals. All animal procedures were approved by the Institutional Animal Care and Use Committee of Creighton University. All efforts were made to minimize pain.

### Materials

Afatinib dimaleate was purchased from Cayman Chemical, USA. Zorifertinib (AZD3759) and AZD5438 were purchased from MedChemExpress, USA. Antibodies used included the following: C-terminal binding protein 2 (mouse anti-CtBP2; BD Transduction Labs, 1:200, catalog no. 612044, RRID:AB_399431), Myosin VIIA (rabbit anti–Myosin VIIA; Proteus Biosciences, 1:250, catalog no. 25-6790, RRID:AB_ 10015251), anti-otoferlin (DSHB 1:500, catalog no. HCS-1, RRID:AB_10804296), anti–green fluorescent protein (anti-GFP; Novus Biologicals, 1:500, catalog no. NB100-1614 RRID:AB_523902), total AKT and ERK (Cell Signaling Technology, catalog nos. 9272S and 4695S; RRID:AB_329827 and RRID:AB_390779, respectively), phospho forms (AKT-S473 and ERK1/2-T202/Y204, Cell Signaling Technology, catalog nos. 9271 and 9101; RRID:AB_329825, RRID:AB_331646, respectively), and β-actin (Sigma-Aldrich, catalog no. A3854, RRID:AB_262011).

### Drug identification using LINCS query

Microarray and RNA-seq transcriptomes from cochlea following noise exposure that are available in the public Gene Expression Omnibus (GEO) database were analyzed using the GEO2R tool of the National Center for Biotechnology Information (NCBI) (www.ncbi.nlm.nih.gov/geo/geo2r/) to identify DEGs between the two groups. Genes with an absolute log FC > 1 were downloaded from each study and analyzed with LINCS databases to identify compounds inducing similar gene expression profiles in various cell lines. The LINCS analysis relies on a subset of the 1,319,138 genetic profiles originally compiled in the L1000 compendium. For each profile, an overlap score between 0 and 1 was given, indicating the fraction of genes that either mimic or reverse the gene set input. With more than 100 identified compounds of interest, we further narrowed down the results of our screen by selecting those compounds with an overlap score >0.1, indicating at least a 10% overlap between the small molecule perturbation from the databases and our gene expression profile.

Three comparison groups were created from the DNA microarray dataset of Gratton *et al*. (*40*). The first experimental group compared transcriptomes of the 129X1/SvJ mouse without noise exposure to the B6.CAST mouse without noise exposure. This group served as our control group (N^−/−^) and identified DEGs that may have conferred resistance to NIHL in the 129X1/SvJ mouse before noise exposure. The second experimental group compares the transcriptomes of the 129X1/SvJ noise-treated mouse to the 129X1/SvJ control mouse and is referred to as 129 N^+/−^. The purpose of this group is to determine which genes may be involved in hearing protection for the 129X1/SvJ following noise exposure. The third experimental group compares the transcriptomes of the B6.CAST noise-treated mouse to B6.CAST control mouse and is referred to as B6 N^+/−^. The purpose of this group is to determine which genes may be involved in noise trauma ototoxicity after noise exposure.

A total of 12,488 DEGs were identified for each of the three experiment groups using GEO2R and were ranked on the basis of *P* value and log FC. DEGs with a log FC > 2 and *P* < 0.05 were considered DEGs of interest. Using this threshold, 92 up-regulated genes and 146 down-regulated genes were found for the N^−/−^ group, 138 up-regulated genes and 24 down-regulated genes were found for the 129 N^+/−^ group, and 109 up-regulated genes and 41 down-regulated genes were found for the B6 N^+/−^ group. DEGs of interest were used for GO pathway analysis using the ShinyGO enrichment analysis program (http://bioinformatics.sdstate.edu/go76/). This program identified 30 biological pathways for each experimental group. Pathways were ranked on the basis of enrichment FDR value and the program identified which of the input genes were significant for each biological pathway.

L1000CDS^2^ analyses were performed using the DEGs from each biological pathway with at least three up-regulated and three down-regulated DEGs of interest. Each L1000CDS^2^ analysis reveals 50 drug perturbations that mimic or reverse the input transcriptome and are ranked on the basis of overlap score. In total, 65 L1000CDS^2^ analyses were performed; 27 analyses from the N^−/−^ Mimic group, 13 analyses from the 129 N^+/−^ Mimic group, and 25 from the B6 N^+/−^ Reverse group. Therefore, 1350 drug perturbations were found that mimic the N^−/−^ DEGs, 650 drug perturbations that mimic the 129 N^+/−^ DEGs, and 1250 drug perturbations that reverse the B6 N^+/−^ DEGs. Drug perturbations with the highest overlap scores were considered significant and filtered the list of significant drug perturbations down to 189 significant drug perturbations from the N^−/−^ Mimic group, 53 significant drug perturbations from 129 N^+/−^ Mimic group, and 173 significant drug perturbations from the B6 N^+/−^ Reverse group. Drug perturbations were further filtered by targeting which drugs target multiple pathways. In total, 83 drug perturbations were found to target multiple pathways between the three experimental groups.

Two experimental groups were created from the bulk RNA-seq dataset of Maeda *et al*. (*41*). The transcriptome of the C57B6 mouse without noise exposure was compared to the C57B6 mouse after noise exposure and identified 939 DEGs. DEGs with a log FC > 2 were considered significant. Of the 939 DEGs, 51 significant up-regulated genes and 222 significant down-regulated genes were identified. In addition, the Maeda group examined significant DEGs that encode transcription factors and identified 9 significant up-regulated genes and 16 significant down-regulated genes. The first experimental group used all 51 up-regulated genes and 222 down-regulated genes as input for an L1000CDS^2^ analysis. The second experimental group used the 9 up-regulated genes and 16 down-regulated genes that encode transcription factors as input for an L1000CDS^2^ analysis. The purpose of these two experiments was to identify drug perturbations that would reverse the transcriptome of the noise-exposed C57B6 mouse. Each of these experiments identified 50 drug perturbations that were ranked on the basis of overlap score for a total of 100 drug perturbations. Of these 100 drug perturbations identified, 43 drug perturbations were also identified from the dataset of Gratton *et al*. (*40*)

For the third dataset from Milon *et al*. (*42*), DEGs for OHCs and supporting cells (6 and 24 hours), marginal, intermediate, basal cells, fibrocytes from the cochlear lateral wall, and the spiral ganglion neurons were used as input for L1000CDS^2^ queries. DEGs with a log FC > 1.2 were considered to be significant. For each cell type, the top 50 drugs ranked according to the cosine distance score were compiled. From the list of 500 drug perturbations, drugs, and mechanism of action classes were compared, and a consensus list was prepared for the three datasets.

### Molecular docking

Structure modeling, docking, and analysis were done using the YASARA package (*88*). For docking dacomitinib and zorifertinib to the EGFR, the crystal structure of the EGFR kinase–afatinib complex was used (PDB id.4G5J). The missing residues 747 to 756 (LREATSPKAN) from the x-ray structure were inserted using the loop modeling option of YASARA. The completed structure was solvated with water molecules in a rectangular box so that the distance between the protein and the box edge was 10 Å. The structure of the solvated system was energy-minimized using the AMBER ff14SB force field (*89*) and then it was subjected to 1-ns molecular dynamics (MD) simulation at 300-K temperature and 1-atm pressure. Using the last frame of the MD trajectory, afatinib was removed from the complex and the structures of dacomitinib and zorifertinib, obtained from https://pubchem.ncbi.nlm.nih.gov, were docked to the receptor using AutoDock VINA (*90*) in YASARA. Molecular contact surface area and contact area color are determined by the ESPPBS method, which includes the implicit water effects.

### Animals and drug administration

#### Zebrafish

*Danio rerio* experimental larvae were obtained by pair mating of adult fish maintained at Creighton University by standard methods approved by the Institutional Animal Care and Use Committee. We used Tg(brn3c:mGFP) expressing a membrane-bound GFP in hair cells (HCs). Experimental fish were maintained at 28.5°C in E3 water [5 mM NaCl, 0.17 mM KCl, 0.33 mM CaCl_2_, and 0.33 nM MgSO_4_ (pH 7.2)]. Animals were cryo-anaesthetized after drug treatment and before fixation. The neuromasts inspected, SO3 and O1–2, were part of the cranial system. In *D. rerio*, while there are two copies of the Egfr receptor gene (Egfra, Egfrb), a single copy of the Egf gene codes for all four ligands. For the EGF pathway KD studies, we used an antisense morpholino oligonucleotide targeting the splice donor site of exon 4 of Egf (*91*, *92*). The morpholino sequence used was 5′-AAGAGAAACCGAGGCTGTACCTTCA-3′. The primer sequences used for Egf expression analysis were egf-forward GACTGCGATGTAAACGCTGA, egf-reverse GCCATTTTTGTGTTGCAATG, β-actin forward CGAGCTGTCTTCCCATCCA, and β-actin reverse TCACCAACGTAGCTGTCTTTCTG. Primers for the downstream molecular targets were previously described in (*93*–*96*).

#### Mice

This study used 7- to 10-week-old FVB/NJ mice obtained from The Jackson Laboratory (Bar Harbor, ME, USA), with an equal number of males and females across the experiments. Conditional EGFR knockout mice were generated by crossing floxed EGFR mouse strain [Egfr^tm1Dwt^ (*97*), stock # 031765-UNC, Mutant Mouse Resource and Research Center] with Pax2-Cre strain (*98*). The mice were on mixed background (CD1, CBA/CaJ, and C57BL/6).

### Zebrafish drug studies

The lateral-line neuromasts of zebrafish are a valuable system for testing the protectivity of compounds against cisplatin toxicity in vivo, as their HCs are considered homologous to those in the mammalian inner ear and are readily accessible to drugs.

To test whether the drugs protect HCs from excitotoxic trauma, we used a zebrafish model that mimics noise damage by exposing fish to the ionotropic glutamate receptor agonist, NMDA previously shown to cause progressive HC loss in zebrafish lateral-line organs. Briefly, 5-dpf larvae were preincubated with 300 μM NMDA for 50 min followed by 2 hours of incubation with the drugs at 0.002 and 0.0183 μM.

Subsequently, animals were transferred to E3 water for 1 hour, fixed in 4% paraformaldehyde (PFA) overnight, and immunostained for otoferlin and GFP. HCs were manually counted using a Zeiss AxioSkop 2 fluorescence microscope with a 40× oil objective. Average HC counts in our studies were obtained from the following three neuromasts: MI1 (middle neuromast 1) and O1 and O2 (otic line). Compounds were then evaluated on efficiency and potency, with the top-rated compounds showing high protection at lower concentrations. For neuromast imaging, samples were analyzed under a Zeiss LSM 700 confocal microscope with an oil immersion objective of 63× (numerical aperture 1.4) and 1.3× digital zoom.

### Noise exposure

FVB/NJ mice (6 to 7 weeks old) were exposed to noise inside a double-walled soundproof booth (IAC Acoustics). Awake mice held individually in small open-walled cylindrical containers inside a reverberant chamber were exposed to octave band noise (8- to 16-kHz at 100-dB SPL) for 2 hours delivered via an exponential horn fitted onto a titanium horn driver (JBL 2426H) and driven by a power amplifier (Crown XTi1000) using RPvdsEx circuit and a RZ6 Multi-I/O processor (Tucker Davis Technologies, Alachua, FL). Before each session, the overall noise level was measured and calibrated at the center and four quadrants of the reverberant chamber using a calibrated 6.35-mm microphone (PCB 377C10). All noise exposures were performed between noon and 2:00 p.m. to minimize differences in circadian variation in sensitivity to noise. Noise exposure level was based on our previously published work (*27*) showing 8- to 16-kHz octave band noise at 100 dB for 2 hours causes 20-dB ABR threshold shift and IHC synaptic ribbon loss.

### Auditory brainstem response

Mice were anesthetized with avertin (5 mg/10 g body weight, ip) and placed on a homeothermic heating pad in conjunction with a rectal temperature probe. Subcutaneous electrodes were inserted at the vertex (reference), posterior to the pinna (active), and over the hip (ground). The acoustical stimulus generation, ABR wave acquisition, equipment control, and data management were performed using the National Instruments PXI system with 6221 and 4461 modules and the EPL Cochlear Function Test Suite (CFTS). Stimuli were presented by a TDT MF1 driver in an open-field configuration placed 10 cm from the left ear of each animal. Tone-pip stimuli of 5-ms duration (0.5-ms rise/fall) at half-octave frequency intervals from 4.0 to 64 kHz were presented at 21/s. At each frequency, an intensity series was presented from 10- to 100-dB SPL in 5-dB incremental steps. A total of 256 responses of alternating stimulus polarity were collected and averaged for each stimulus level. Evoked-response signal was amplified 10,000× (Grass P5-11 bio-amplifier) and band-pass–filtered (0.3 to 3 kHz) before digitization. ABR threshold was determined as the lowest stimulus level that produced a detectable ABR waveform (waves 1 to 5) that could be visualized. ABR threshold shifts were calculated by subtraction of the pre-exposure thresholds from the postexposure thresholds.

### Distortion product otoacoustic emissions

The primary tones f1 and f2 were generated and shaped using EPL CFTS software and the NI PXI system. The two primary tones were presented using two TDT MF1 speakers in closed-field configurations. The primary tones were delivered using a custom probe insert attached to a low-noise ER10B^+^ microphone (Etymotic Research, USA). DPOAEs were recorded in the form of level/frequency functions; f2/f1 was fixed at 1.2, with the level of the f2 (L2)10 dB less than the f1 level (L1). The f2 stimuli were presented at 5.6 to 32 kHz at half-octave intervals. At each f2 frequency, L2 was varied between 65- and 5-dB SPL at 10-dB steps. The 2f1-f2 DPOAE amplitude and surrounding noise floor were extracted by offline analysis. DPOAE threshold was defined as the L1 level that produced emission at 2f1–f2 with an amplitude of 0-dB SPL. The average noise floor was −25-dB SPL across frequencies.

### Sample preparation and immunofluorescence labeling

After the final ABR and DPOAE measurements, the mice were transcardially perfused with 4% PFA in 0.1 M phosphate buffer. After perfusion, cochleae were isolated and postfixed in 4% PFA in 0.1 M phosphate buffer for 2 hours at room temperature. After fixation, the cochleae were decalcified in 120 mM EDTA for 2 to 3 days. Each cochlea was microdissected into three pieces and a cochlear frequency map was computed on the basis of 3D reconstruction of the sensory epithelium for OHCs and presynaptic ribbon counts. Dissected pieces were permeabilized using 0.02% Triton X-100 for 15 min, washed three times in phosphate-buffered saline, and preincubated for 1 hour in blocking buffer (10% normal goat serum) at room temperature. Cochlear pieces were incubated with CtBP2 or Myosin VIIa, with matching secondary antibodies (Alexa Fluor 488, 546; Life Technologies, USA). 4′,6-Diamidino-2-phenylindole was used for nuclear staining (DAPI; Thermo Fisher Scientific, 1:1000). Stained cochlear pieces were mounted on slides with Fluoromount-G medium (SouthernBiotech, USA) and cover-slipped.

### Quantification of synaptic ribbons

Following the frequency map computation, cochlear structures were located in relevant frequency regions. Using a confocal microscope (Zeiss LSM 700), OHC and IHC zones were both imaged with a 63×, numerical aperture 1.4 with 1.0× digital zoom. For IHC ribbon synapse quantification, 3D (*x*-*y*-*z* axis) images were scanned with the 1.3× digital zoom at 63×. Each immunolabeled presynaptic CtBP2 puncta was counted as a ribbon synapse. Synaptic ribbons of 10 to 14 consecutive IHCs distributed within the 8- to10-, 16- to 22-, and 32- to 40-kHz frequency regions were counted. The CtBP2 (presynaptic) puncta were counted using Imaris 9.5 (Oxford Instruments, Abingdon, UK) using the approach described by Fogarty *et al*. (*99*). The average spot diameter was set to 0.45 μm, and only CtBP2 puncta found within the surface of the IHC were included. The synaptic ribbons in the normal cochleae were also calculated using the same method to serve as control comparison samples.

### Western blotting

For the immunoblot studies, animals were administered afatinib, zorifertinib, or the vehicle 1 day before and immediately after the noise exposure. Animals were euthanized either 30 min after the noise exposure and the cochleae were isolated and processed in lysis buffer (radioimmunoprecipitation assay, Thermo Fisher Scientific, 89901) containing protease inhibitors. Fifteen to 30 μg of protein were used for the immunoblot experiments. Membranes were blocked with 3% of the milk-blocking solution and incubated with the primary antibodies overnight at 4°C. After several washes and incubation with the secondary antibody, membranes were developed using a ChemiBlot system (Bio-Rad). Membranes were stripped and reprobed for the phosphorylated forms. β-Actin was used as the loading control.

### Kinome analyses

#### Identification of significant differential AKT and ERK family kinase activity

Twenty-four AKT and 25 ERK kinase family putative target peptides were identified by the KRSA package (*100*). Log_2_ FCs in peptide activity were calculated by comparing noise, zorifertinib, or noise + zorifertinib to control. For each peptide, an average of triplicates was used per condition. Two-way analysis of variance (ANOVA) was used to identify significant differences between groups (****P < 0.0001 and ***P < 0.001), with results presented as means ± SD.

#### Methods omnibus for the PamGene kinome array

##### Overview

The PamGene platform is a well-established, highly cited, microarray technology for multiplex kinase activity profiling (*84*, *85*, *101*, *102*).

##### Hardware

Pamstation12 and PamChip4: The PamGene12 kinome array is a peptide array–based platform that facilitates the unbiased detection of kinase activity by STK or protein tyrosine kinase (PTK) (*84*, *85*, *101*–*104*). The STK and PTK PamGene12 chips have 144 and 196 reporter peptides, respectively. Each spot has approximately 300,000 copies of the same peptide printed on it. Phosphorylation is detected in real time. Fluorescent antibodies are applied against phosphorylated residues; fluorescent intensity is a proxy for the extent of reporter peptide phosphorylation (fig. S5). Altered kinase activity can be directly measured. For example, phosphorylation by PKA on the STK chip is concordant with its activity in solution (*101*). Each peptide chip has four wells, and three chips can be run at the same time. Thus, there are up to 12 samples for each “run” on the array.

##### Chip coverage

Of the about 500 kinases in the human genome (*105*, *106*), 245 of 376 (65%) Ser/Thr and 89 of 93 (96%) Tyr kinases can be mapped to the STK and PTK chips, respectively. The chips also map about 18 of 21 (86%) dual specificity kinases, covering about 72% of the entire kinome. The STK chip covers similar amounts of low (52%), medium (65%), and high (65%) abundance protein kinases in neurons [based on Brainseq neuron database (www.brainrnaseq.org/)] and has a sensitivity for detection into the picogram range for many kinases.

#### Kinome array protocols

##### Data generation

Samples are prepared according to the protocols provided by PamGene Corp (https://pamgene.com/ps12/). The catalytic activity and stability of kinases are controlled by the addition of protease and phosphatase inhibitors. Peptide phosphorylation is monitored during the incubation with an assay mixture, by taking images every 5 min for 60 min at exposure lengths of 5, 25, and 100 ms, allowing real-time recording of the reaction kinetics. Various internal control tests have been performed by PamGene International to ensure the sensitivity of the assay. Chip-to-chip and run-to-run technical variations (coefficient of variability) are <9% and <15%, respectively. To account for technical variation between runs, an internal control sample may be added to account for between-run variability.

##### Preliminary data processing

The primary output from PamStation12 is images from the Evolve kinetic image capture software. These images are then pre-processed to quantify the activity at each peptide level using PamGene’s BioNavigator software (https://pamgene.com/wp-content/uploads/2020/09/BioNavigator-User-Manual-vs2.3-2020.pdf). Before proceeding to activity analysis, all peptides that appear inactive (Raw signal <= 5) are removed from the analysis. The dynamic range of the raw signal intensities is typically 0 to 3000. Linear regression slope of the signal intensity as a function of exposure time is used to represent the peptide phosphorylation intensity for downstream comparative analyses averaged across the biological replicates. This is done to increase the dynamic range of the measurements. The signal ratio between case and control samples is used to calculate FC values. Peptides with an FC of at least 15% (i.e., FC > 1.15 or FC < 0.85) are considered differentially phosphorylated to use KRSA. This threshold was chosen on the basis of previous reports that suggest small changes in kinase activity are sufficient to trigger biologically relevant changes (*103*, *104*, *107*). Peptides that had a very low signal or an R^2^ of less than 0.90 during the corresponding linear regression were considered undetectable or non-linear in the post-wash phase and were excluded from subsequent analyses.

##### Assessment of upstream kinases

Peptides spotted on the array (and in general) may be phosphorylated by more than one kinases and in many cases several different kinases. The use of two different types of chips, one for STK and one for Tyr kinases (STK) provides a starting point for the assignment of kinases. There are four different software packages that may be deployed for the assignment of upstream kinases. All of them rely, to varying extents, on publicly available mapping databases. Each has strengths and weaknesses, some of which are discussed below.

##### Upstream kinase analysis

This package was developed by the Pamgene Corp (s’-Hertogenbosch, Netherlands). UKA is integrated into the manufacturer’s BioNavigator software and their recommended method. This method relies on a curated database of kinase-substrate interactions created by the PamGene Corp. It takes the raw output from the PamStation as input. It then filters low-intensity peptides and scales the entire dataset to the range of 0 to 100. It then calculates a “kinase score” for each kinase and reports the ones with the highest score. Advantages include (i) providing results for specific kinases (as opposed to families) and (ii) a low false positive rate compared to other packages. One putative weakness is that it may be too stringent for discovery-based experiments.

##### Kinome random sampling analyzer

The package was developed by the Cognitive Disorders Research Laboratory (CDRL) at the College of Medicine and Life Sciences (COMLS) University of Toledo, led by R. McCullumsmith (*100*). The data generated from the kinome array experiment and the mapping of the PamChip file are used as input to the algorithm. Once selected, peptides are filtered out using advancement criteria, including the signal intensity at maximum exposure time and the R2 values of the linear regression of signal intensity as a function of exposure time. At the end of this step, a list of filtered peptides moves forward to the next step of the analysis.

##### Curation of the database of upstream kinases

KRSA relies on a curated database of upstream kinases for the peptides present on the array. Protein kinases predicted to act on phosphorylation sites within the array peptide sequences were identified using GPS 3.0 and Kinexus Phosphonet (Kinexus Bioinformatics) (*108*–*110*). These programs provide predictions for serine-threonine kinases targeting peptide sequences ordered by likelihood of binding. The union of the highest-ranked five kinases in Kinexus and kinases with scores more than two times the prediction threshold in GPS 3.0 were considered predicted kinases for each peptide and used in KRSA analysis (*101*). This list was combined with kinases shown in the literature to act on the phosphorylation sites of the peptides via PhosphoELM (http://phospho.elm.eu.org) and PhosphoSite Plus (www.phosphosite.org).

#### Presentation of the data in KRSA

##### Heatmaps

Heatmaps are generated from the signal intensity data. The selection of peptides is based on the quality control criteria explained above. The values on the heatmap are the slopes of the linear models of signal intensity as a function of the exposure time.

##### Violin plots

Violin plots showcase the distribution of the signal intensity of significant peptides on a per-group basis.

##### Waterfall plots

Waterfall plots are generated from the *z*-score values for each kinase. These values are generated on a chip-by-chip basis and then averaged across the three. The plot shows the distribution of these three points and a red dot shows the mean *z*-score value.

##### Kinase enrichment analysis 3

KEA3 is an upstream kinase assignment method developed by the Maayan laboratory (https://maayanlab.cloud/kea3/) that relies on the known kinase protein interactions and kinase-substrate interaction data and associated coexpression and co-occurrence data (*111*). The KEA3 web server takes a set of phosphorylated proteins and their fold-change value as input and returns the putative upstream kinases using the database and looking for statistically significant overrepresentation of kinases.

##### PTM signature enrichment analysis

PTM-SEA (*112*, *113*) is an application developed by the Broad Institute to identify putative upstream kinases. PTM-SEA is a modified form of the single sample Gene Set Enrichment Analysis (ssGSEA) with the underlying database built on top of PTMSigDB (*112*). The software runs on the R Programming language. It takes in files in the Gene Cluster Text format which has the peptides and log FC values in a specified format. The output from PTM-SEA is a list of putative upstream kinases that can phosphorylate each site.

##### Integration of upstream kinase assignments across packages

All the tools above use different methods to assign upstream kinases. This necessitates the use of an integration system to identify consensus upstream kinases across datasets. For this purpose, we use the software Creedenzymatic (*114*). The Creedenzymatic analysis takes in the results from at least two of the 4 analyses mentioned above and then generates a consensus figure with kinases deconvolved and ranked based on their presence in the results.

#### Perilymph sampling and quantitative mass spectrometry

FVB mice (6 to 8 weeks old) were administered zorifertinib (15 mg/kg) via oral gavage. Inner ear perilymph fluid was collected before and after (30 min, 1 hour, 2 hours, 4 hours, and 6 hours) the drug treatment. One-microliter samples were collected from the posterior-most, extracranial portion of the posterior semi-circular canal (*115*) and diluted 50-fold with 0.1% formic acid in water. Samples were frozen for later analysis and quantified via our LC-MS/MS in the Mass Spectrometry Core. Mice with vehicle treatment only were used as negative controls. Samples were spiked with a standard amount of 1 μg/ml IS (crizotinib). Twenty microliters of the diluted sample were injected in a Vanquish UPLC through a Waters Acquity BEH 18C 1.7 μm × 2.1 μm × 50 mm column coupled to a Q Exactive quadrupole mass spectrometer with electrospray ionization (ESI) interface. The column was maintained at 40°C. The mobile phase consisted of eluent A (0.1% formic acid in water) and eluent B (acetonitrile) at a flow rate of 0.2 ml/min with the following gradient: 0 to 1 min – 5%B; 1 to 4 min – 40%B; 4 to 6 min – 50%B;0.6 to 8 min – 65%B, 8 to 10 mins 100%B; 10 to 22 mins – 5%B. ESI conditions were spray voltage, 3.9 kV; capillary temp, 320°C. The PRM inclusion list was 460.153 (zorifertinib) and 450.13 (IS), and detection was run in positive po. All acquisition and analysis of data was done using Xcalibur software (Thermo Fisher Scientific, MA, USA).

### Statistical analysis

All statistical analyses and graphical visualization were performed in GraphPad Prism v9.x (GraphPad, MA, USA). Comparisons between the treatment groups for ABR and DPOAEs were performed using two-way ANOVA followed by Holm-Šidák or Bonferroni post hoc tests. A paired Student’s t test was used for the comparison of the CtBP2 puncta between experimental groups. ABR/DPOAE thresholds and CtBP2 puncta counts were determined by an independent observer who was blinded to the treatment condition. For zebrafish quantification studies one-way ANOVA was performed followed by a Dunnet posthoc test. Statistical significance was set at *P* ≤ 0.05. Unless stated otherwise, the results are expressed as means ± SEM.

## References

[1] J. S. Gordon, S. E. Griest, E. J. Thielman, K. F. Carlson, W. J. Helt, M. S. Lewis, C. Blankenship, D. Austin, S. M. Theodoroff, J. A. Henry, Audiologic characteristics in a sample of recently-separated military veterans: The noise outcomes in servicemembers epidemiology study (NOISE Study). Hear. Res. 349, 21–30 (2017). 27913314 10.1016/j.heares.2016.11.014

[2] J. T. Nelson, A. A. Swan, B. Swiger, M. Packer, M. J. Pugh, Hearing testing in the U.S. Department of Defense: Potential impact on Veterans Affairs hearing loss disability awards. Hear. Res. 349, 13–20 (2017). 27768901 10.1016/j.heares.2016.10.005

[3] A. A. Swan, J. T. Nelson, B. Swiger, C. A. Jaramillo, B. C. Eapen, M. Packer, M. J. Pugh, Prevalence of hearing loss and tinnitus in Iraq and Afghanistan Veterans: A chronic effects of neurotrauma consortium study. Hear. Res. 349, 4–12 (2017). 28153668 10.1016/j.heares.2017.01.013

[4] K. Yankaskas, Prelude: Noise-induced tinnitus and hearing loss in the military. Hear. Res. 295, 3–8 (2013). 22575206 10.1016/j.heares.2012.04.016

[5] E. Kerns, E. A. Masterson, C. L. Themann, G. M. Calvert, Cardiovascular conditions, hearing difficulty, and occupational noise exposure within US industries and occupations. Am. J. Ind. Med. 61, 477–491 (2018). 29537072 10.1002/ajim.22833 PMC6897488

[6] S. Tak, R. R. Davis, G. M. Calvert, Exposure to hazardous workplace noise and use of hearing protection devices among US workers—NHANES, 1999–2004. Am. J. Ind. Med. 52, 358–371 (2009). 19267354 10.1002/ajim.20690

[7] S. D. Nash, K. J. Cruickshanks, R. Klein, B. E. Klein, F. J. Nieto, G. H. Huang, J. S. Pankow, T. S. Tweed, The prevalence of hearing impairment and associated risk factors: The beaver dam offspring study. Arch. Otolaryngol. Head Neck Surg. 137, 432–439 (2011). 21339392 10.1001/archoto.2011.15 PMC3096733

[8] L. M. Haile, A. U. Orji, K. M. Reavis, P. S. Briant, K. M. Lucas, F. Alahdab, T. W. Barnighausen, A. W. Bell, C. Cao, X. Dai, S. I. Hay, G. Heidari, I. M. Karaye, T. R. Miller, A. H. Mokdad, E. Mostafavi, Z. S. Natto, S. Pawar, J. Rana, A. Seylani, J. A. Singh, J. Wei, L. Yang, K. L. Ong, J. D. Steinmetz; GBD 2019 USA Hearing Loss Collaborators, Hearing loss prevalence, years lived with disability, and hearing aid use in the United States from 1990 to 2019: Findings from the global burden of disease study. Ear Hear. 45, 257–267 (2024). 37712826 10.1097/AUD.0000000000001420 PMC10718207

[9] K. Graydon, C. Waterworth, H. Miller, H. Gunasekera, Global burden of hearing impairment and ear disease. J. Laryngol. Otol. 133, 18–25 (2019). 30047343 10.1017/S0022215118001275

[10] Centers for Disease Control and Prevention, Severe hearing impairment among military veterans—United States, 2010. MMWR Morb. Mortal. Wkly. Rep. 60, 955–958 (2011). 21775950

[11] K. D. Yankaskas, J. M. Komrower, Military and industrial performance: The critical role of noise controls. Int. J. Audiol. 58, S74–S80 (2019). 30589388 10.1080/14992027.2018.1534013

[12] R. L. Neitzel, T. K. Swinburn, M. S. Hammer, D. Eisenberg, Economic impact of hearing loss and reduction of noise-induced hearing loss in the United States. J. Speech Lang. Hear. Res. 60, 182–189 (2017). 28056138 10.1044/2016_JSLHR-H-15-0365

[13] P. Dawes, R. Emsley, K. J. Cruickshanks, D. R. Moore, H. Fortnum, M. Edmondson-Jones, A. McCormack, K. J. Munro, Hearing loss and cognition: The role of hearing AIDS, social isolation and depression. PLOS ONE 10, e0119616 (2015). 25760329 10.1371/journal.pone.0119616 PMC4356542

[14] F. Panza, M. Lozupone, R. Sardone, P. Battista, M. Piccininni, V. Dibello, M. La Montagna, R. Stallone, P. Venezia, A. Liguori, G. Giannelli, A. Bellomo, A. Greco, A. Daniele, D. Seripa, N. Quaranta, G. Logroscino, Sensorial frailty: Age-related hearing loss and the risk of cognitive impairment and dementia in later life. Ther. Adv. Chronic Dis. 10, 2040622318811000 (2019). 31452865 10.1177/2040622318811000 PMC6700845

[15] H. E. Whitson, A. Cronin-Golomb, K. J. Cruickshanks, G. C. Gilmore, C. Owsley, J. E. Peelle, G. Recanzone, A. Sharma, B. Swenor, K. Yaffe, F. R. Lin, American geriatrics society and national institute on aging bench-to-bedside conference: Sensory impairment and cognitive decline in older adults. J. Am. Geriatr. Soc. 66, 2052–2058 (2018). 30248173 10.1111/jgs.15506 PMC6410371

[16] D. C. Kohrman, G. Wan, L. Cassinotti, G. Corfas, Hidden hearing loss: A disorder with multiple etiologies and mechanisms. Cold Spring Harb. Perspect. Med. 10, a035493 (2020). 30617057 10.1101/cshperspect.a035493 PMC6612463

[17] Y. Wang, K. Hirose, M. C. Liberman, Dynamics of noise-induced cellular injury and repair in the mouse cochlea. J. Assoc. Res. Otolaryngol. 3, 248–268 (2002). 12382101 10.1007/s101620020028 PMC3202415

[18] D. Henderson, E. C. Bielefeld, K. C. Harris, B. H. Hu, The role of oxidative stress in noise-induced hearing loss. Ear Hear. 27, 1–19 (2006). 16446561 10.1097/01.aud.0000191942.36672.f3

[19] R. A. Hazlitt, J. Min, J. Zuo, Progress in the development of preventative drugs for cisplatin-induced hearing loss. J. Med. Chem. 61, 5512–5524 (2018). 29361217 10.1021/acs.jmedchem.7b01653 PMC6043375

[20] L. P. Rybak, C. A. Whitworth, D. Mukherjea, V. Ramkumar, Mechanisms of cisplatin-induced ototoxicity and prevention. Hear. Res. 226, 157–167 (2007). 17113254 10.1016/j.heares.2006.09.015

[21] R. A. Hazlitt, T. Teitz, J. D. Bonga, J. Fang, S. Diao, L. Iconaru, L. Yang, A. N. Goktug, D. G. Currier, T. Chen, Z. Rankovic, J. Min, J. Zuo, Development of second-generation CDK2 inhibitors for the prevention of cisplatin-induced hearing loss. J. Med. Chem. 61, 7700–7709 (2018). 30091915 10.1021/acs.jmedchem.8b00669 PMC6443376

[22] M. A. Ingersoll, E. A. Malloy, L. E. Caster, E. M. Holland, Z. Xu, M. Zallocchi, D. Currier, H. Liu, D. Z. Z. He, J. Min, T. Chen, J. Zuo, T. Teitz, BRAF inhibition protects against hearing loss in mice. Sci. Adv. 6, eabd0561 (2020). 33268358 10.1126/sciadv.abd0561 PMC7821884

[23] N. J. Ingham, S. A. Pearson, V. E. Vancollie, V. Rook, M. A. Lewis, J. Chen, A. Buniello, E. Martelletti, L. Preite, C. C. Lam, F. D. Weiss, Z. Powis, P. Suwannarat, C. J. Lelliott, S. J. Dawson, J. K. White, K. P. Steel, Mouse screen reveals multiple new genes underlying mouse and human hearing loss. PLoS Biol. 17, e3000194 (2019). 30973865 10.1371/journal.pbio.3000194 PMC6459510

[24] H. W. Lim, K. Pak, A. F. Ryan, A. Kurabi, Screening mammalian cochlear hair cells to identify critical processes in aminoglycoside-mediated damage. Front. Cell. Neurosci. 12, 179 (2018). 30013464 10.3389/fncel.2018.00179 PMC6036173

[25] H. C. Ou, L. L. Cunningham, S. P. Francis, C. S. Brandon, J. A. Simon, D. W. Raible, E. W. Rubel, Identification of FDA-approved drugs and bioactives that protect hair cells in the zebrafish (Danio rerio) lateral line and mouse (Mus musculus) utricle. J. Assoc. Res. Otolaryngol. 10, 191–203 (2009). 19241104 10.1007/s10162-009-0158-y PMC2674201

[26] M. Ryals, R. J. Morell, D. Martin, E. T. Boger, P. Wu, D. W. Raible, L. L. Cunningham, The inner ear heat shock transcriptional signature identifies compounds that protect against aminoglycoside ototoxicity. Front. Cell. Neurosci. 12, 445 (2018). 30532693 10.3389/fncel.2018.00445 PMC6265442

[27] T. Teitz, J. Fang, A. N. Goktug, J. D. Bonga, S. Diao, R. A. Hazlitt, L. Iconaru, M. Morfouace, D. Currier, Y. Zhou, R. A. Umans, M. R. Taylor, C. Cheng, J. Min, B. Freeman, J. Peng, M. F. Roussel, R. Kriwacki, R. K. Guy, T. Chen, J. Zuo, CDK2 inhibitors as candidate therapeutics for cisplatin- and noise-induced hearing loss. J. Exp. Med. 215, 1187–1203 (2018). 29514916 10.1084/jem.20172246 PMC5881471

[28] F. Iorio, T. Rittman, H. Ge, M. Menden, J. Saez-Rodriguez, Transcriptional data: A new gateway to drug repositioning? Drug Discov. Today 18, 350–357 (2013). 22897878 10.1016/j.drudis.2012.07.014 PMC3625109

[29] J. Li, S. Zheng, B. Chen, A. J. Butte, S. J. Swamidass, Z. Lu, A survey of current trends in computational drug repositioning. Brief. Bioinform. 17, 2–12 (2016). 25832646 10.1093/bib/bbv020 PMC4719067

[30] J. Lamb, E. D. Crawford, D. Peck, J. W. Modell, I. C. Blat, M. J. Wrobel, J. Lerner, J. P. Brunet, A. Subramanian, K. N. Ross, M. Reich, H. Hieronymus, G. Wei, S. A. Armstrong, S. J. Haggarty, P. A. Clemons, R. Wei, S. A. Carr, E. S. Lander, T. R. Golub, The connectivity map: Using gene-expression signatures to connect small molecules, genes, and disease. Science 313, 1929–1935 (2006). 17008526 10.1126/science.1132939

[31] A. Subramanian, R. Narayan, S. M. Corsello, D. D. Peck, T. E. Natoli, X. Lu, J. Gould, J. F. Davis, A. A. Tubelli, J. K. Asiedu, D. L. Lahr, J. E. Hirschman, Z. Liu, M. Donahue, B. Julian, M. Khan, D. Wadden, I. C. Smith, D. Lam, A. Liberzon, C. Toder, M. Bagul, M. Orzechowski, O. M. Enache, F. Piccioni, S. A. Johnson, N. J. Lyons, A. H. Berger, A. F. Shamji, A. N. Brooks, A. Vrcic, C. Flynn, J. Rosains, D. Y. Takeda, R. Hu, D. Davison, J. Lamb, K. Ardlie, L. Hogstrom, P. Greenside, N. S. Gray, P. A. Clemons, S. Silver, X. Wu, W. N. Zhao, W. Read-Button, X. Wu, S. J. Haggarty, L. V. Ronco, J. S. Boehm, S. L. Schreiber, J. G. Doench, J. A. Bittker, D. E. Root, B. Wong, T. R. Golub, A next generation connectivity map: L1000 platform and the first 1,000,000 profiles. Cell 171, 1437–1452.e17 (2017).29195078

[32] Q. Duan, S. P. Reid, N. R. Clark, Z. Wang, N. F. Fernandez, A. D. Rouillard, B. Readhead, S. R. Tritsch, R. Hodos, M. Hafner, M. Niepel, P. K. Sorger, J. T. Dudley, S. Bavari, R. G. Panchal, A. Ma'ayan, L1000CDS^2^: LINCS L1000 characteristic direction signatures search engine. NPJ Syst. Biol. Appl. 2, 16015 (2016). 28413689 10.1038/npjsba.2016.15 PMC5389891

[33] M. M. Moasser, Targeting the function of the HER2 oncogene in human cancer therapeutics. Oncogene 26, 6577–6592 (2007). 17486079 10.1038/sj.onc.1210478 PMC3071580

[34] B. Malgrange, B. Rogister, P. P. Lefebvre, C. Mazy-Servais, A. A. Welcher, C. Bonnet, R. Y. Hsu, J. M. Rigo, T. R. Van De Water, G. Moonen, Expression of growth factors and their receptors in the postnatal rat cochlea. Neurochem. Res. 23, 1133–1138 (1998). 9704604 10.1023/a:1020724506337

[35] A. Zine, F. de Ribaupierre, Tissue-specific levels and cellular distribution of epidermal growth factor receptors within control and neomycin-damaged neonatal rat Organ of Corti. J. Neurobiol. 38, 313–322 (1999). 10022575

[36] A. Zine, M. Nyffeler, F. de Ribaupierre, Spatial expression patterns of epidermal growth factor receptor gene transcripts in the postnatal mammalian cochlea. Hear. Res. 141, 19–27 (2000). 10713492 10.1016/s0378-5955(99)00203-8

[37] M. Zhang, D. Ding, R. Salvi, Expression of heregulin and ErbB/Her receptors in adult chinchilla cochlear and vestibular sensory epithelium. Hear. Res. 169, 56–68 (2002). 12121740 10.1016/s0378-5955(02)00339-8

[38] P. M. White, J. S. Stone, A. K. Groves, N. Segil, EGFR signaling is required for regenerative proliferation in the cochlea: Conservation in birds and mammals. Dev. Biol. 363, 191–200 (2012). 22230616 10.1016/j.ydbio.2011.12.035 PMC3288574

[39] J. Zhang, Q. Wang, D. Abdul-Aziz, J. Mattiacio, A. S. B. Edge, P. M. White, ERBB2 signaling drives supporting cell proliferation in vitro and apparent supernumerary hair cell formation in vivo in the neonatal mouse cochlea. Eur. J. Neurosci. 48, 3299–3316 (2018). 30270571 10.1111/ejn.14183 PMC6234075

[40] M. A. Gratton, A. Eleftheriadou, J. Garcia, E. Verduzco, G. K. Martin, B. L. Lonsbury-Martin, A. E. Vazquez, Noise-induced changes in gene expression in the cochleae of mice differing in their susceptibility to noise damage. Hear. Res. 277, 211–226 (2011). 21187137 10.1016/j.heares.2010.12.014 PMC3098916

[41] Y. Maeda, S. Kariya, K. Uraguchi, J. Takahara, S. Fujimoto, A. Sugaya, K. Nishizaki, Immediate changes in transcription factors and synaptic transmission in the cochlea following acoustic trauma: A gene transcriptome study. Neurosci. Res. 165, 6–13 (2021). 32417196 10.1016/j.neures.2020.05.001

[42] B. Milon, E. D. Shulman, K. S. So, C. R. Cederroth, E. L. Lipford, M. Sperber, J. B. Sellon, H. Sarlus, G. Pregernig, B. Shuster, Y. Song, S. Mitra, J. Orvis, Z. Margulies, Y. Ogawa, C. Shults, D. A. Depireux, A. T. Palermo, B. Canlon, J. Burns, R. Elkon, R. Hertzano, A cell-type-specific atlas of the inner ear transcriptional response to acoustic trauma. Cell Rep. 36, 109758 (2021). 34592158 10.1016/j.celrep.2021.109758 PMC8709734

[43] K. Stepnik, W. Kukula-Koch, In silico studies on triterpenoid saponins permeation through the blood-brain barrier combined with postmortem research on the brain tissues of mice affected by astragaloside IV administration. Int. J. Mol. Sci. 21, 2534 (2020). 32260588 10.3390/ijms21072534 PMC7177733

[44] J. Chen, K. Hill, S. H. Sha, Inhibitors of histone deacetylases attenuate noise-induced hearing loss. J. Assoc. Res. Otolaryngol. 17, 289–302 (2016). 27095478 10.1007/s10162-016-0567-7 PMC4940287

[45] H. Wang, G. Yang, D. Sun, B. Wang, H. Chen, M. Chen, B. Zhu, Histone deacetylase 2 polymorphisms associated with noise-induced hearing loss in Chinese workers. Environ. Sci. Pollut. Res. Int. 28, 38254–38262 (2021). 33733414 10.1007/s11356-021-13486-5

[46] F. Depreux, L. Czech, H. Young, C. P. Richter, Y. Zhou, D. S. Whitlon, Statins protect mice from high-decibel noise-induced hearing loss. Biomed. Pharmacother. 163, 114674 (2023). 37435721 10.1016/j.biopha.2023.114674

[47] H. Shen, B. Zhang, J. H. Shin, D. Lei, Y. Du, X. Gao, Q. Wang, K. K. Ohlemiller, J. Piccirillo, J. Bao, Prophylactic and therapeutic functions of T-type calcium blockers against noise-induced hearing loss. Hear. Res. 226, 52–60 (2007). 17291698 10.1016/j.heares.2006.12.011 PMC1903349

[48] T. Yamashita, F. Zheng, D. Finkelstein, Z. Kellard, R. Carter, C. D. Rosencrance, K. Sugino, J. Easton, C. Gawad, J. Zuo, High-resolution transcriptional dissection of in vivo Atoh1-mediated hair cell conversion in mature cochleae identifies Isl1 as a co-reprogramming factor. PLOS Genet. 14, e1007552 (2018). 30063705 10.1371/journal.pgen.1007552 PMC6086484

[49] Z. Xu, S. Tu, C. Pass, Y. Zhang, H. Liu, J. Diers, Y. Fu, D. Z. Z. He, J. Zuo, Profiling mouse cochlear cell maturation using 10× genomics single-cell transcriptomics. Front. Cell. Neurosci. 16, 962106 (2022). 36060279 10.3389/fncel.2022.962106 PMC9434313

[50] C. R. Hume, M. Kirkegaard, E. C. Oesterle, ErbB expression: The mouse inner ear and maturation of the mitogenic response to heregulin. J. Assoc. Res. Otolaryngol. 4, 422–443 (2003). 14690060 10.1007/s10162-002-3008-8 PMC3202727

[51] K. Stankovic, C. Rio, A. Xia, M. Sugawara, J. C. Adams, M. C. Liberman, G. Corfas, Survival of adult spiral ganglion neurons requires erbB receptor signaling in the inner ear. J. Neurosci. 24, 8651–8661 (2004). 15470130 10.1523/JNEUROSCI.0733-04.2004 PMC6729966

[52] L. Sheets, Excessive activation of ionotropic glutamate receptors induces apoptotic hair-cell death independent of afferent and efferent innervation. Sci. Rep. 7, 41102 (2017). 28112265 10.1038/srep41102 PMC5255535

[53] N. Hakuba, K. Koga, K. Gyo, S. I. Usami, K. Tanaka, Exacerbation of noise-induced hearing loss in mice lacking the glutamate transporter GLAST. J. Neurosci. 20, 8750–8753 (2000). 11102482 10.1523/JNEUROSCI.20-23-08750.2000 PMC6773045

[54] J. L. Puel, J. Ruel, C. Gervais d'Aldin, R. Pujol, Excitotoxicity and repair of cochlear synapses after noise-trauma induced hearing loss. Neuroreport 9, 2109–2114 (1998). 9674603 10.1097/00001756-199806220-00037

[55] Q. Wang, S. H. Green, Functional role of neurotrophin-3 in synapse regeneration by spiral ganglion neurons on inner hair cells after excitotoxic trauma in vitro. J. Neurosci. 31, 7938–7949 (2011). 21613508 10.1523/JNEUROSCI.1434-10.2011 PMC3132175

[56] S. Bahl, H. Ling, N. P. N. Acharige, I. Santos-Barriopedro, M. K. H. Pflum, E. Seto, EGFR phosphorylates HDAC1 to regulate its expression and anti-apoptotic function. Cell Death Dis. 12, 469 (2021). 33976119 10.1038/s41419-021-03697-6 PMC8113371

[57] T. M. Brand, M. Iida, C. Li, D. L. Wheeler, The nuclear epidermal growth factor receptor signaling network and its role in cancer. Discov. Med. 12, 419–432 (2011). 22127113 PMC3305885

[58] K. Xu, H.-K. G. Shu, EGFR activation results in enhanced cyclooxygenase-2 expression through p38 mitogen-activated protein kinase-dependent activation of the Sp1/Sp3 transcription factors in human gliomas. Cancer Res. 67, 6121-9 (2007). 17616668 10.1158/0008-5472.CAN-07-0141

[59] S. G. Kujawa, M. C. Liberman, Adding insult to injury: Cochlear nerve degeneration after “temporary” noise-induced hearing loss. J. Neurosci. 29, 14077–14085 (2009). 19906956 10.1523/JNEUROSCI.2845-09.2009 PMC2812055

[60] L. D. Liberman, J. Suzuki, M. C. Liberman, Dynamics of cochlear synaptopathy after acoustic overexposure. J. Assoc. Res. Otolaryngol. 16, 205–219 (2015). 25676132 10.1007/s10162-015-0510-3 PMC4368657

[61] Y. Maeda, K. Fukushima, R. Omichi, S. Kariya, K. Nishizaki, Time courses of changes in phospho- and total- MAP kinases in the cochlea after intense noise exposure. PLOS ONE 8, e58775 (2013). 23484051 10.1371/journal.pone.0058775 PMC3590164

[62] I. Meltser, Y. Tahera, B. Canlon, Differential activation of mitogen-activated protein kinases and brain-derived neurotrophic factor after temporary or permanent damage to a sensory system. Neuroscience 165, 1439–1446 (2010). 19925854 10.1016/j.neuroscience.2009.11.025

[63] O. Selivanova, J. Brieger, U. R. Heinrich, W. Mann, Akt and c-Jun N-terminal kinase are regulated in response to moderate noise exposure in the cochlea of guinea pigs. ORL J. Otorhinolaryngol Relat. Spec. 69, 277–282 (2007). 17565230 10.1159/000103871

[64] S. Jamesdaniel, B. Hu, M. H. Kermany, H. Jiang, D. Ding, D. Coling, R. Salvi, Noise induced changes in the expression of p38/MAPK signaling proteins in the sensory epithelium of the inner ear. J. Proteomics 75, 410–424 (2011). 21871588 10.1016/j.jprot.2011.08.007 PMC3225708

[65] A. S. Imami, S. M. O'Donovan, J. F. Creeden, X. Wu, H. Eby, C. B. McCullumsmith, K. Uvnas-Moberg, R. E. McCullumsmith, E. Andari, Oxytocin’s anti-inflammatory and proimmune functions in COVID-19: A transcriptomic signature-based approach. Physiol. Genomics 52, 401–407 (2020). 32809918 10.1152/physiolgenomics.00095.2020 PMC7877479

[66] R. Shukla, N. D. Henkel, K. Alganem, A. R. Hamoud, J. Reigle, R. S. Alnafisah, H. M. Eby, A. S. Imami, J. F. Creeden, S. A. Miruzzi, J. Meller, R. E. McCullumsmith, Signature-based approaches for informed drug repurposing: Targeting CNS disorders. Neuropsychopharmacology 46, 116–130 (2021). 32604402 10.1038/s41386-020-0752-6 PMC7688959

[67] C. R. Sullivan, C. A. Mielnik, S. M. O'Donovan, A. J. Funk, E. Bentea, E. A. DePasquale, K. Alganem, Z. Wen, V. Haroutunian, P. Katsel, A. J. Ramsey, J. Meller, R. E. McCullumsmith, Connectivity analyses of bioenergetic changes in schizophrenia: Identification of novel treatments. Mol. Neurobiol. 56, 4492–4517 (2019). 30338483 10.1007/s12035-018-1390-4 PMC7584383

[68] N. S. Jahchan, J. T. Dudley, P. K. Mazur, N. Flores, D. Yang, A. Palmerton, A. F. Zmoos, D. Vaka, K. Q. Tran, M. Zhou, K. Krasinska, J. W. Riess, J. W. Neal, P. Khatri, K. S. Park, A. J. Butte, J. Sage, A drug repositioning approach identifies tricyclic antidepressants as inhibitors of small cell lung cancer and other neuroendocrine tumors. Cancer Discov. 3, 1364–1377 (2013). 24078773 10.1158/2159-8290.CD-13-0183 PMC3864571

[69] S. J. Xiao, X. C. Zhu, H. Deng, W. P. Zhou, W. Y. Yang, L. K. Yuan, J. Y. Zhang, S. Tian, L. Xu, L. Zhang, H. M. Xia, Gene expression profiling coupled with Connectivity Map database mining reveals potential therapeutic drugs for Hirschsprung disease. J. Pediatr. Surg. 53, 1716–1721 (2018). 29605259 10.1016/j.jpedsurg.2018.02.060

[70] L. Zhang, W. Kang, X. Lu, S. Ma, L. Dong, B. Zou, Weighted gene co-expression network analysis and connectivity map identifies lovastatin as a treatment option of gastric cancer by inhibiting HDAC2. Gene 681, 15–25 (2019). 30266498 10.1016/j.gene.2018.09.040

[71] T. Asano, S. Chelvanambi, J. L. Decano, M. C. Whelan, E. Aikawa, M. Aikawa, In silico drug screening approach using L1000-based connectivity map and its application to COVID-19. Front. Cardiovasc. Med. 9, 842641 (2022). 35402570 10.3389/fcvm.2022.842641 PMC8989014

[72] A. S. Imami, R. E. McCullumsmith, S. M. O'Donovan, Strategies to identify candidate repurposable drugs: COVID-19 treatment as a case example. Transl. Psychiatry 11, 591 (2021). 34785660 10.1038/s41398-021-01724-w PMC8594646

[73] R. S. Wang, J. Loscalzo, Repurposing drugs for the treatment of COVID-19 and its cardiovascular manifestations. Circ. Res. 132, 1374–1386 (2023). 37167362 10.1161/CIRCRESAHA.122.321879 PMC10171294

[74] C. G. Le Prell, Otoprotectants: From research to clinical application. Semin. Hear. 40, 162–176 (2019). 31036993 10.1055/s-0039-1684045 PMC6486371

[75] K. Fernandez, K. K. Spielbauer, A. Rusheen, L. Wang, T. G. Baker, S. Eyles, L. L. Cunningham, Lovastatin protects against cisplatin-induced hearing loss in mice. Hear. Res. 389, 107905 (2020). 32062294 10.1016/j.heares.2020.107905 PMC7080598

[76] D. Lei, X. Gao, P. Perez, K. K. Ohlemiller, C. C. Chen, K. P. Campbell, A. Y. Hood, J. Bao, Anti-epileptic drugs delay age-related loss of spiral ganglion neurons via T-type calcium channel. Hear. Res. 278, 106–112 (2011). 21640179 10.1016/j.heares.2011.05.010 PMC3152691

[77] Boehringer Ingelheim Pharmaceuticals, Pharmacology Review NDA# 201292, Center for Drug Evaluation and Research (U.S. Food and Drug Administration, 2013).

[78] L. V. Sequist, J. C. Yang, N. Yamamoto, K. O'Byrne, V. Hirsh, T. Mok, S. L. Geater, S. Orlov, C. M. Tsai, M. Boyer, W. C. Su, J. Bennouna, T. Kato, V. Gorbunova, K. H. Lee, R. Shah, D. Massey, V. Zazulina, M. Shahidi, M. Schuler, Phase III study of afatinib or cisplatin plus pemetrexed in patients with metastatic lung adenocarcinoma with EGFR mutations. J. Clin. Oncol. 31, 3327–3334 (2013). 23816960 10.1200/JCO.2012.44.2806

[79] M. Kim, J. K. Laramy, A. S. Mohammad, S. Talele, J. Fisher, J. N. Sarkaria, W. F. Elmquist, Brain distribution of a panel of epidermal growth factor receptor inhibitors using cassette dosing in wild-type and Abcb1/Abcg2-deficient mice. Drug Metab. Dispos. 47, 393–404 (2019). 30705084 10.1124/dmd.118.084210 PMC6408736

[80] Q. Zeng, J. Wang, Z. Cheng, K. Chen, P. Johnstrom, K. Varnas, D. Y. Li, Z. F. Yang, X. Zhang, Discovery and evaluation of clinical candidate AZD3759, a potent, oral active, central nervous system-penetrant, epidermal growth factor receptor tyrosine kinase inhibitor. J. Med. Chem. 58, 8200–8215 (2015). 26313252 10.1021/acs.jmedchem.5b01073

[81] M. J. Ahn, D. W. Kim, B. C. Cho, S. W. Kim, J. S. Lee, J. S. Ahn, T. M. Kim, C. C. Lin, H. R. Kim, T. John, S. Kao, J. W. Goldman, W. C. Su, R. Natale, S. Rabbie, B. Harrop, P. Overend, Z. Yang, J. C. Yang, Activity and safety of AZD3759 in EGFR-mutant non-small-cell lung cancer with CNS metastases (BLOOM): A phase 1, open-label, dose-escalation and dose-expansion study. Lancet Respir. Med. 5, 891–902 (2017). 29056570 10.1016/S2213-2600(17)30378-8

[82] K. R. Henry, Cochlear damage resulting from exposure to four different octave bands of noise at three ages. Behav. Neurosci. 98, 107–117 (1984). 6696793 10.1037//0735-7044.98.1.107

[83] L. Di, H. Rong, B. Feng, Demystifying brain penetration in central nervous system drug discovery. Miniperspective. J. Med. Chem. 56, 2–12 (2013). 23075026 10.1021/jm301297f

[84] R. Arsenault, P. Griebel, S. Napper, Peptide arrays for kinome analysis: New opportunities and remaining challenges. Proteomics 11, 4595–4609 (2011). 22002874 10.1002/pmic.201100296

[85] A. Baharani, B. Trost, A. Kusalik, S. Napper, Technological advances for interrogating the human kinome. Biochem. Soc. Trans. 45, 65–77 (2017). 28202660 10.1042/BST20160163

[86] N. Jongkamonwiwat, M. A. Ramirez, S. Edassery, A. C. Y. Wong, J. Yu, T. Abbott, K. Pak, A. F. Ryan, J. N. Savas, Noise exposures causing hearing loss generate proteotoxic stress and activate the proteostasis network. Cell Rep. 33, 108431 (2020). 33238128 10.1016/j.celrep.2020.108431 PMC7722268

[87] R. A. Dobie, L. E. Humes, Commentary on the regulatory implications of noise-induced cochlear neuropathy. Int. J. Audiol. 56, 74–78 (2017). 27849127 10.1080/14992027.2016.1255359

[88] E. Krieger, G. Vriend, New ways to boost molecular dynamics simulations. J. Comput. Chem. 36, 996–1007 (2015). 25824339 10.1002/jcc.23899 PMC6680170

[89] F. O. Kok, M. Shin, C. W. Ni, A. Gupta, A. S. Grosse, A. van Impel, B. C. Kirchmaier, J. Peterson-Maduro, G. Kourkoulis, I. Male, D. F. DeSantis, S. Sheppard-Tindell, L. Ebarasi, C. Betsholtz, S. Schulte-Merker, S. A. Wolfe, N. D. Lawson, Reverse genetic screening reveals poor correlation between morpholino-induced and mutant phenotypes in zebrafish. Dev. Cell 32, 97–108 (2015). 25533206 10.1016/j.devcel.2014.11.018 PMC4487878

[90] O. Trott, A. J. Olson, AutoDock Vina: Improving the speed and accuracy of docking with a new scoring function, efficient optimization, and multithreading. J. Comput. Chem. 31, 455–461 (2010). 19499576 10.1002/jcc.21334 PMC3041641

[91] J. A. Laisney, I. Braasch, R. B. Walter, S. Meierjohann, M. Schartl, Lineage-specific co-evolution of the Egf receptor/ligand signaling system. BMC Evol. Biol. 10, 27 (2010). 20105326 10.1186/1471-2148-10-27 PMC2834686

[92] B. Pruvot, Y. Cure, J. Djiotsa, A. Voncken, M. Muller, Developmental defects in zebrafish for classification of EGF pathway inhibitors. Toxicol. Appl. Pharmacol. 274, 339–349 (2014). 24262764 10.1016/j.taap.2013.11.006

[93] K. T. Duffy, M. F. McAleer, W. R. Davidson, L. Kari, C. Kari, C. G. Liu, S. A. Farber, K. C. Cheng, J. R. Mest, E. Wickstrom, A. P. Dicker, U. Rodeck, Coordinate control of cell cycle regulatory genes in zebrafish development tested by cyclin D1 knockdown with morpholino phosphorodiamidates and hydroxyprolyl-phosphono peptide nucleic acids. Nucleic Acids Res. 33, 4914–4921 (2005). 16284195 10.1093/nar/gki799 PMC1199556

[94] Y. He, D. Tang, W. Li, R. Chai, H. Li, Histone deacetylase 1 is required for the development of the zebrafish inner ear. Sci. Rep. 6, 16535 (2016). 26832938 10.1038/srep16535 PMC4735278

[95] T. O. Ishikawa, K. J. Griffin, U. Banerjee, H. R. Herschman, The zebrafish genome contains two inducible, functional cyclooxygenase-2 genes. Biochem. Biophys. Res. Commun. 352, 181–187 (2007). 17112475 10.1016/j.bbrc.2006.11.007 PMC1764854

[96] H. Y. Jeon, H. Lee, Depletion of Aurora-A in zebrafish causes growth retardation due to mitotic delay and p53-dependent cell death. FEBS J. 280, 1518–1530 (2013). 23351126 10.1111/febs.12153

[97] T. C. Lee, D. W. Threadgill, Generation and validation of mice carrying a conditional allele of the epidermal growth factor receptor. Genesis 47, 85–92 (2009). 19115345 10.1002/dvg.20464 PMC9060398

[98] T. Ohyama, A. K. Groves, Generation of Pax2-Cre mice by modification of a Pax2 bacterial artificial chromosome. Genesis 38, 195–199 (2004). 15083520 10.1002/gene.20017

[99] M. J. Fogarty, L. A. Hammond, R. Kanjhan, M. C. Bellingham, P. G. Noakes, A method for the three-dimensional reconstruction of Neurobiotin-filled neurons and the location of their synaptic inputs. Front. Neural Circuits 7, 153 (2013). 24101895 10.3389/fncir.2013.00153 PMC3787200

[100] E. A. K. DePasquale, K. Alganem, E. Bentea, N. Nawreen, J. L. McGuire, T. Tomar, F. Naji, R. Hilhorst, J. Meller, R. E. McCullumsmith, KRSA: An R package and R Shiny web application for an end-to-end upstream kinase analysis of kinome array data. PLOS ONE 16, e0260440 (2021). 34919543 10.1371/journal.pone.0260440 PMC8682895

[101] E. Bentea, E. A. K. Depasquale, S. M. O'Donovan, C. R. Sullivan, M. Simmons, J. H. Meador-Woodruff, Y. Zhou, C. Xu, B. Bai, J. Peng, H. Song, G. L. Ming, J. Meller, Z. Wen, R. E. McCullumsmith, Kinase network dysregulation in a human induced pluripotent stem cell model of DISC1 schizophrenia. Mol. Omics 15, 173–188 (2019). 31106784 10.1039/c8mo00173a PMC6563817

[102] R. Hilhorst, L. Houkes, A. van den Berg, R. Ruijtenbeek, Peptide microarrays for detailed, high-throughput substrate identification, kinetic characterization, and inhibition studies on protein kinase A. Anal. Biochem. 387, 150–161 (2009). 19344656 10.1016/j.ab.2009.01.022

[103] J. L. McGuire, E. A. Depasquale, A. J. Funk, S. M. O'Donnovan, K. Hasselfeld, S. Marwaha, J. H. Hammond, V. Hartounian, J. H. Meador-Woodruff, J. Meller, R. E. McCullumsmith, Abnormalities of signal transduction networks in chronic schizophrenia. NPJ Schizophr. 3, 30 (2017). 28900113 10.1038/s41537-017-0032-6 PMC5595970

[104] C. R. Dorsett, J. L. McGuire, T. L. Niedzielko, E. A. DePasquale, J. Meller, C. L. Floyd, R. E. McCullumsmith, Traumatic brain injury induces alterations in cortical glutamate uptake without a reduction in glutamate transporter-1 protein expression. J. Neurotrauma 34, 220–234 (2017). 27312729 10.1089/neu.2015.4372 PMC5198172

[105] G. Manning, D. B. Whyte, R. Martinez, T. Hunter, S. Sudarsanam, The protein kinase complement of the human genome. Science 298, 1912–1934 (2002). 12471243 10.1126/science.1075762

[106] G. Manning, Genomic overview of Protein Kinases, in *WormBook* (The C. elegans Research Community, 2005). 10.1895/wormbook.1.60.1 PMC4780929 18050405

[107] J. A. Appuhamy, W. A. Nayananjalie, E. M. England, D. E. Gerrard, R. M. Akers, M. D. Hanigan, Effects of AMP-activated protein kinase (AMPK) signaling and essential amino acids on mammalian target of rapamycin (mTOR) signaling and protein synthesis rates in mammary cells. J. Dairy Sci. 97, 419–429 (2014). 24183687 10.3168/jds.2013-7189

[108] Y. Xue, Z. Liu, J. Cao, Q. Ma, X. Gao, Q. Wang, C. Jin, Y. Zhou, L. Wen, J. Ren, GPS 2.1: Enhanced prediction of kinase-specific phosphorylation sites with an algorithm of motif length selection. Protein Eng. Des. Sel. 24, 255–260 (2011). 21062758 10.1093/protein/gzq094

[109] C. Wang, H. Xu, S. Lin, W. Deng, J. Zhou, Y. Zhang, Y. Shi, D. Peng, Y. Xue, GPS 5.0: An update on the prediction of kinase-specific phosphorylation sites in proteins. Genom. Proteom. Bioinform. 18, 72–80 (2020). 10.1016/j.gpb.2020.01.001 PMC7393560 32200042

[110] Y. Xue, F. Zhou, M. Zhu, K. Ahmed, G. Chen, X. Yao, GPS: A comprehensive www server for phosphorylation sites prediction. Nucleic Acids Res. 33, W184–W187 (2005). 15980451 10.1093/nar/gki393 PMC1160154

[111] M. V. Kuleshov, Z. Xie, A. B. K. London, J. Yang, J. E. Evangelista, A. Lachmann, I. Shu, D. Torre, A. Ma'ayan, KEA3: Improved kinase enrichment analysis via data integration. Nucleic Acids Res. 49, W304–W316 (2021). 34019655 10.1093/nar/gkab359 PMC8265130

[112] K. Krug, P. Mertins, B. Zhang, P. Hornbeck, R. Raju, R. Ahmad, M. Szucs, F. Mundt, D. Forestier, J. Jane-Valbuena, H. Keshishian, M. A. Gillette, P. Tamayo, J. P. Mesirov, J. D. Jaffe, S. A. Carr, D. R. Mani, A curated resource for phosphosite-specific signature analysis. Mol. Cell. Proteomics 18, 576–593 (2019). 30563849 10.1074/mcp.TIR118.000943 PMC6398202

[113] J. Cox, M. Mann, MaxQuant enables high peptide identification rates, individualized p.p.b.-range mass accuracies and proteome-wide protein quantification. Nat. Biotechnol. 26, 1367–1372 (2008). 19029910 10.1038/nbt.1511

[114] Khaled, A. S. Imami, J. Creeden, CogDisResLab/creedenzymatic: V 5.0.0 Version Reset. Zenodo (2022); 10.5281/zenodo.6363766.

[115] K. Hirose, J. J. Hartsock, S. Johnson, P. Santi, A. N. Salt, Systemic lipopolysaccharide compromises the blood-labyrinth barrier and increases entry of serum fluorescein into the perilymph. J. Assoc. Res. Otolaryngol. 15, 707–719 (2014). 24952083 10.1007/s10162-014-0476-6 PMC4164684

[116] F. A. Wolf, P. Angerer, F. J. Theis, SCANPY: Large-scale single-cell gene expression data analysis. Genome Biol. 19, 15 (2018). 29409532 10.1186/s13059-017-1382-0 PMC5802054

