# Supplementary material for: In silico transcriptome screens identify epidermal growth factor receptor inhibitors as therapeutics for noise-induced hearing loss
Source: Sci Adv. 2024 Jun 19;10(25):eadk2299. doi: 10.1126/sciadv.adk2299 (PMC11186505; doi:10.1126/sciadv.adk2299)
Supplement: Supplementary file 1 — Figs. S1 to S17 Legend for table S1 [file sciadv.adk2299_sm.pdf]

Science Advances
AAAS

Supplementary Materials for

In silico transcriptome screens identify epidermal growth factor receptor inhibitors as therapeutics for noise-induced hearing loss

Sarath Vijayakumar *et al.*

Corresponding author: Jian Zuo, jianzuo@tingtherapeutics.com

Sci. Adv. **10**, eadk2299 (2024)
DOI: 10.1126/sciadv.adk2299

The PDF file includes:

Figs. S1 to S17
Legend for table S1

Other Supplementary Material for this manuscript includes the following:

Table S1

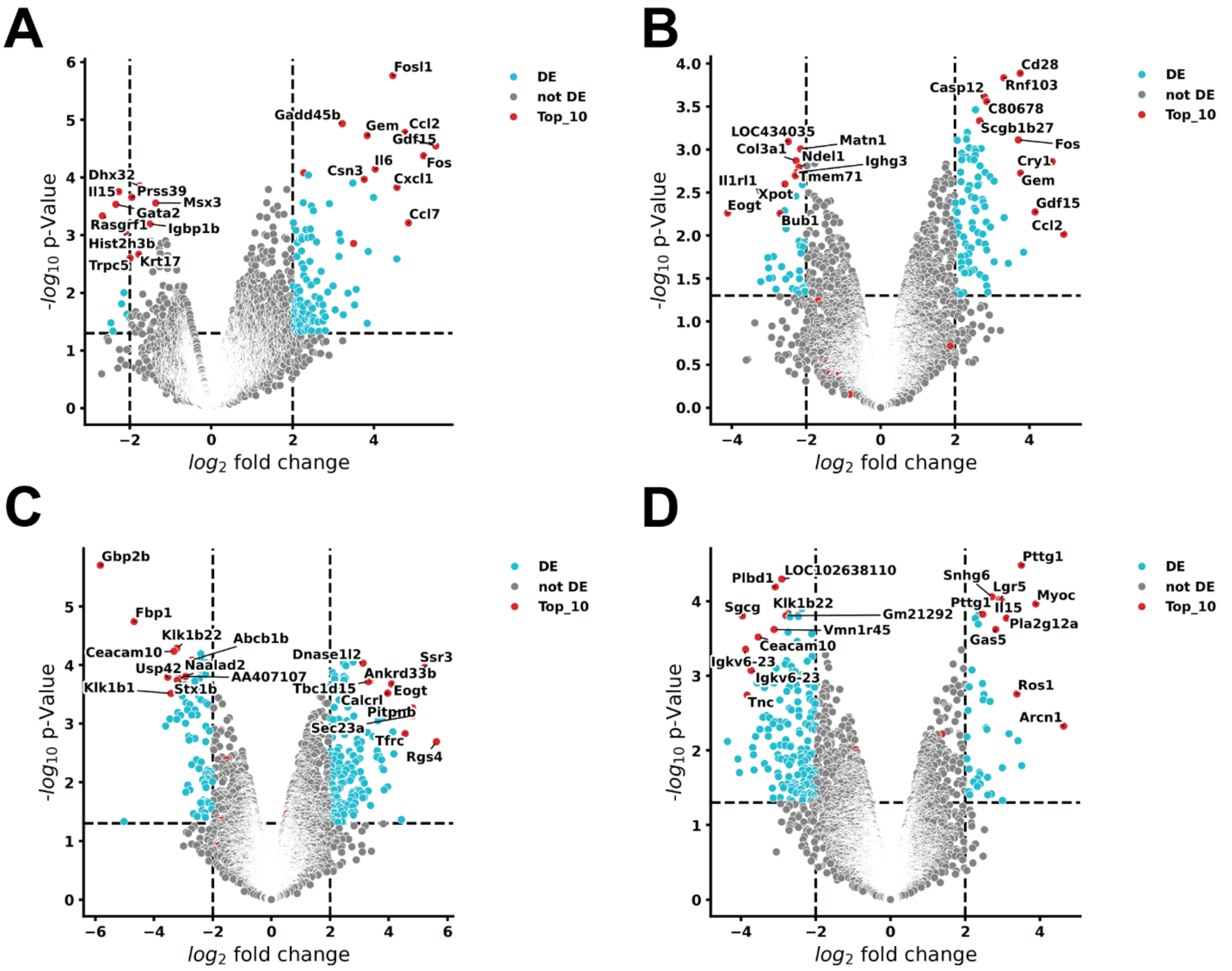

Fig. S1. Differentially expressed genes from Gratton et al. (40) A) 129X1 control (no noise exposure) vs after noise exposure B) B6.CAST control vs. after noise exposure C) 129X1 vs. B6.CAST controls (no noise exposure) D) 129X1 vs B6.CAST after noise exposure. Genes that are above the cut-off thresholds (|$\log_2$FC| > 2 and p-value < 0.05 are shown in cyan. The top 10 genes from the list are shown in red. Vertical dashed lines represent $\log_2$FC 2, -2, horizontal dashed line represent -$\log_{10}$ p-value of 0.05.

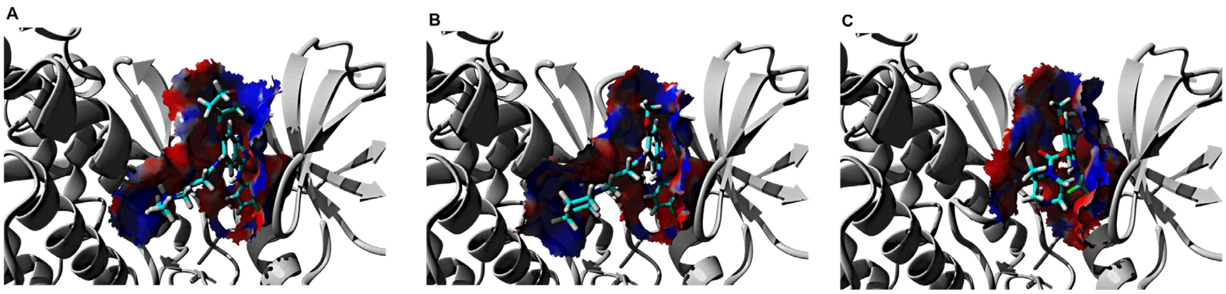

Fig. S2. EGFR kinase inhibitors docked to the receptor binding site. A, Afatinib; **B,** Dacomitinib; **C**, Zorifertinib. Cartoon representation of EGFR is in grey color; inhibitor molecules are in stick representation. Interacting molecular surface area between the EGFR and inhibitors are colored by the electrostatic potential between the protein and ligand; red and blue colors represent the negative and positive potential respectively. The main structural differences among these inhibitors appear at the crotonamide side chain which can be correlated to EGFR signaling differences.

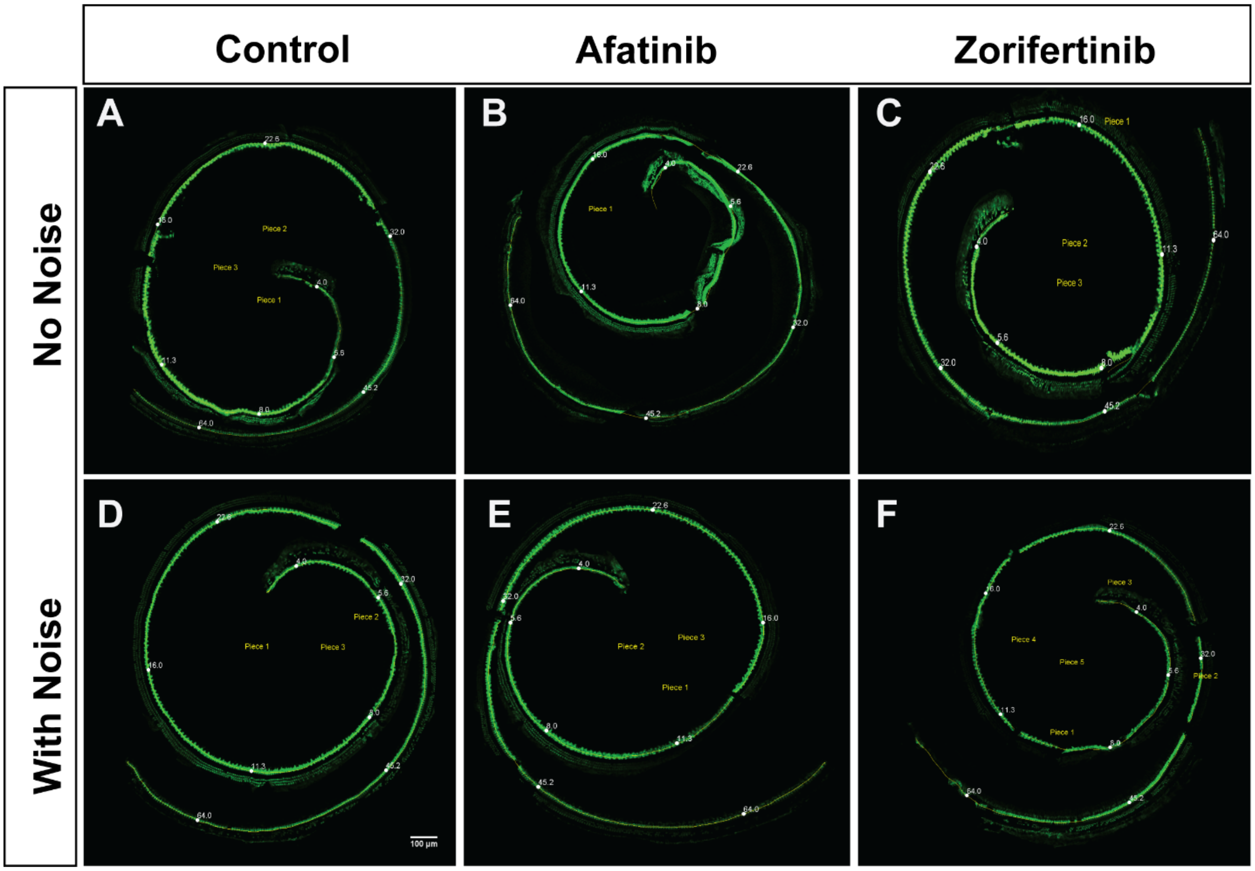

Fig. S3. Moderate-level noise exposure produces cochlear synaptopathy without hair cell loss in FVB mice. Representative images of cochlear whole mounts labeled with Myosin 7A (green) from the different treatment groups do not show outer or inner hair cell loss 14 days after exposure to 100 dB SPL 8 – 18 kHz octave band noise (D-F) when compared to groups that did not receive noise exposure (A-C). Cochlear place frequency mapping is shown along the length of the cochlea. The scale bar in D is 100μm applies to all the images.

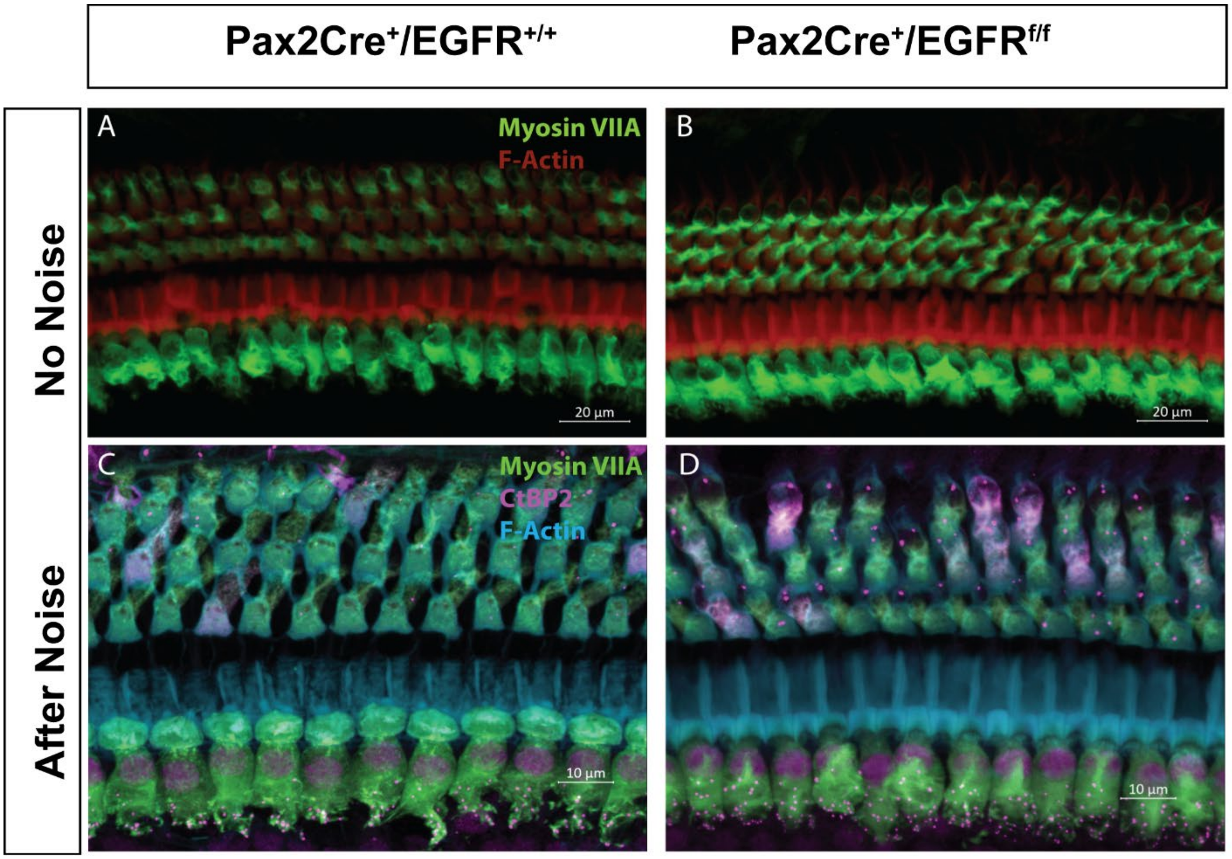

Fig. S4. Conditional knockout of EGFR protects against noise-induced cochlear synatopathy in mice. (**A**) Representative maximum intensity projections of the cochlea's 16-22 kHz region from Pax2Cre^{+}/EGFR$^{+/+}$ (wildtype) and Pax2Cre^{+}/EGFR$^{f/f}$ (homozygous mutant) mice. Hair cells were labeled using Myosin VIIA (green), and phalloidin for F-Actin (red). No obvious difference in hair cell morphology is seen between the two genotypes (**B**) EGFR knockout protects against noise-induced cochlear synaptopathy, resulting in less CtBP2 puncta loss in the homozygous mice compared to wildtype. Hair cells were labeled using Myosin VIIA (green), CtBP2 (magenta) and F-Actin (cyan). Scale bar = 20µm for A, B and 10µm for C, D.

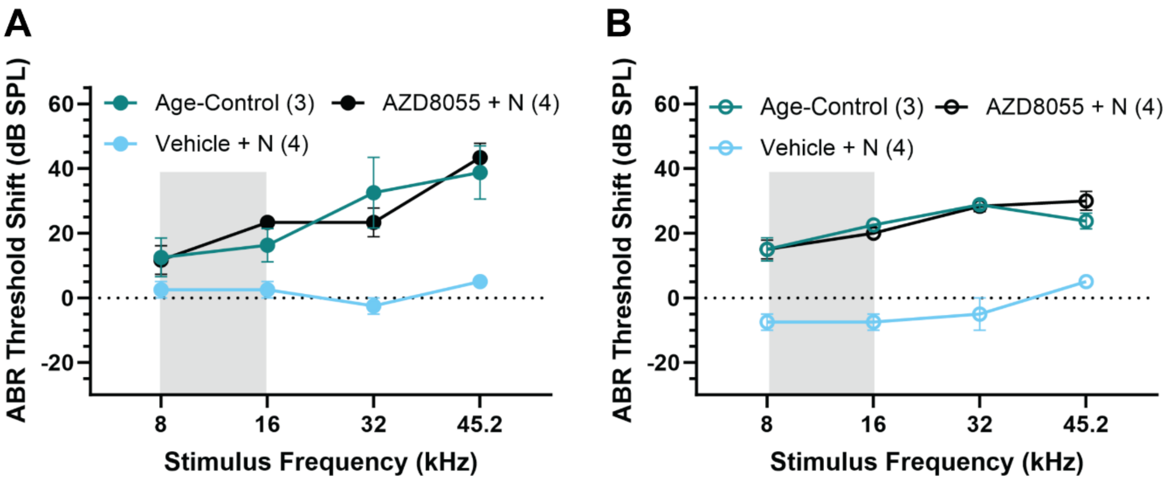

Fig. S5. **AZD-8055 did not protect against noise-induced hearing loss in mice.** Adult FVB mice (4-7 weeks old) were exposed to noise trauma (8-16kHz noise band at 100 dB SPL for 2hrs.) indicated by the shaded box in the figures. **(A)** ABR threshold shifts 1 day after the noise exposure. Animals treated with 4 doses of AZD-8055 10 mg/kg/day IP (black) did show significant differences in the threshold shifts at all tested frequencies when compared to those treated with the vehicle (light blue). **(B)** ABR threshold shifts 2 weeks after the noise exposure. Animals treated with 4 doses of AZD-8055 10 mg/kg/day IP (black) did not show significant differences in the threshold shifts at all tested frequencies when compared to those treated with the vehicle (light blue). ABR threshold shifts measured on days 1 and 14 remain elevated for AZD-8055 and vehicle-treated groups compared to the age-matched controls. Age-matched controls that were not exposed to any treatment (noise/drug/vehicle) showed minimal threshold shifts at all tested frequencies. Data are presented as mean ± SEM, n=3-4/group. “+N” indicates with noise exposure.

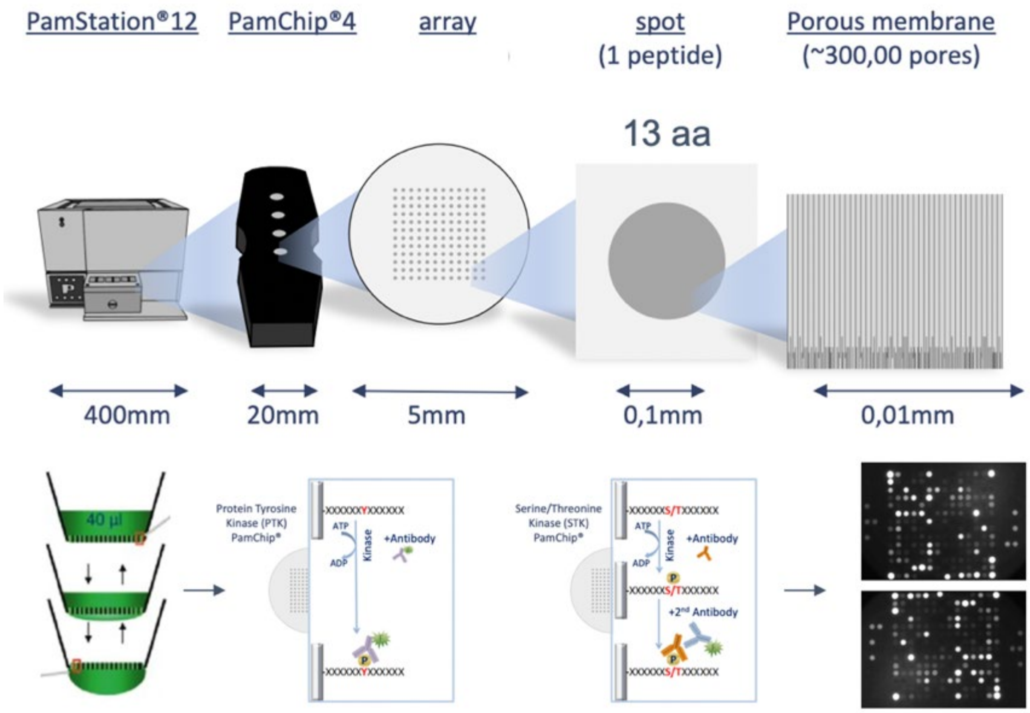

Fig. S6. PamGene Platform Workflow. The arrays are spotted with reporter peptides, including controls, coupled to an activated aluminum oxide surface to create a 3-D structure facilitating interactions. During an experiment, the array is incubated with lysates of cells or tissue. The active kinases in the sample will phosphorylate their target on the array. Generic fluorescent labeled antibodies that recognize phosphorylated residues are used to visualize the phosphorylation in real time. This figure was reprinted with permission from PamGene International B.V.

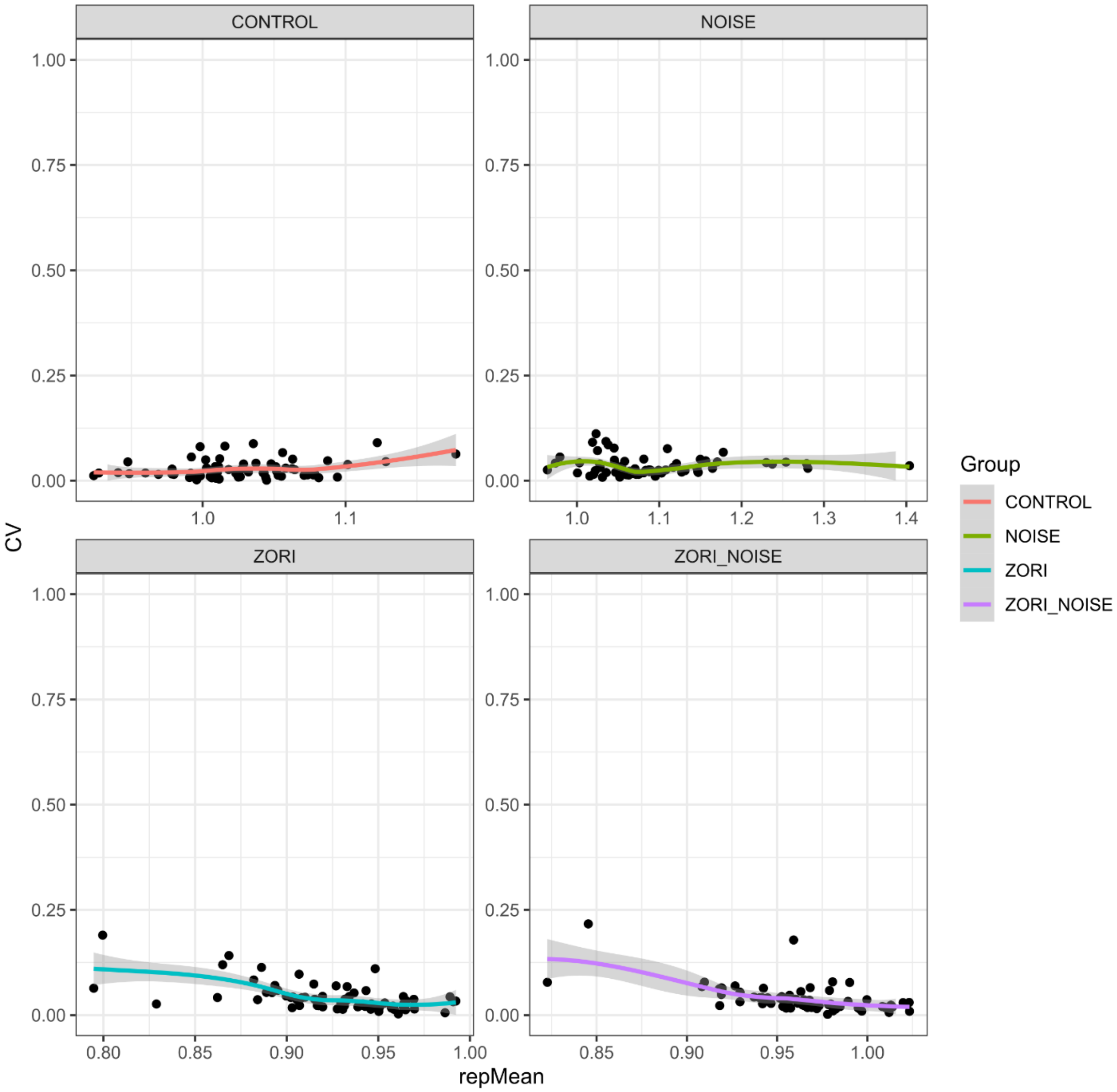

Fig. S7. Coefficient of variation (CVs) of normalized signal intensities for each reporter peptide across control, zorifertinib (Zori), noise, and zorifertinib plus noise groups. Data show low variability across dozens of reporter peptides.

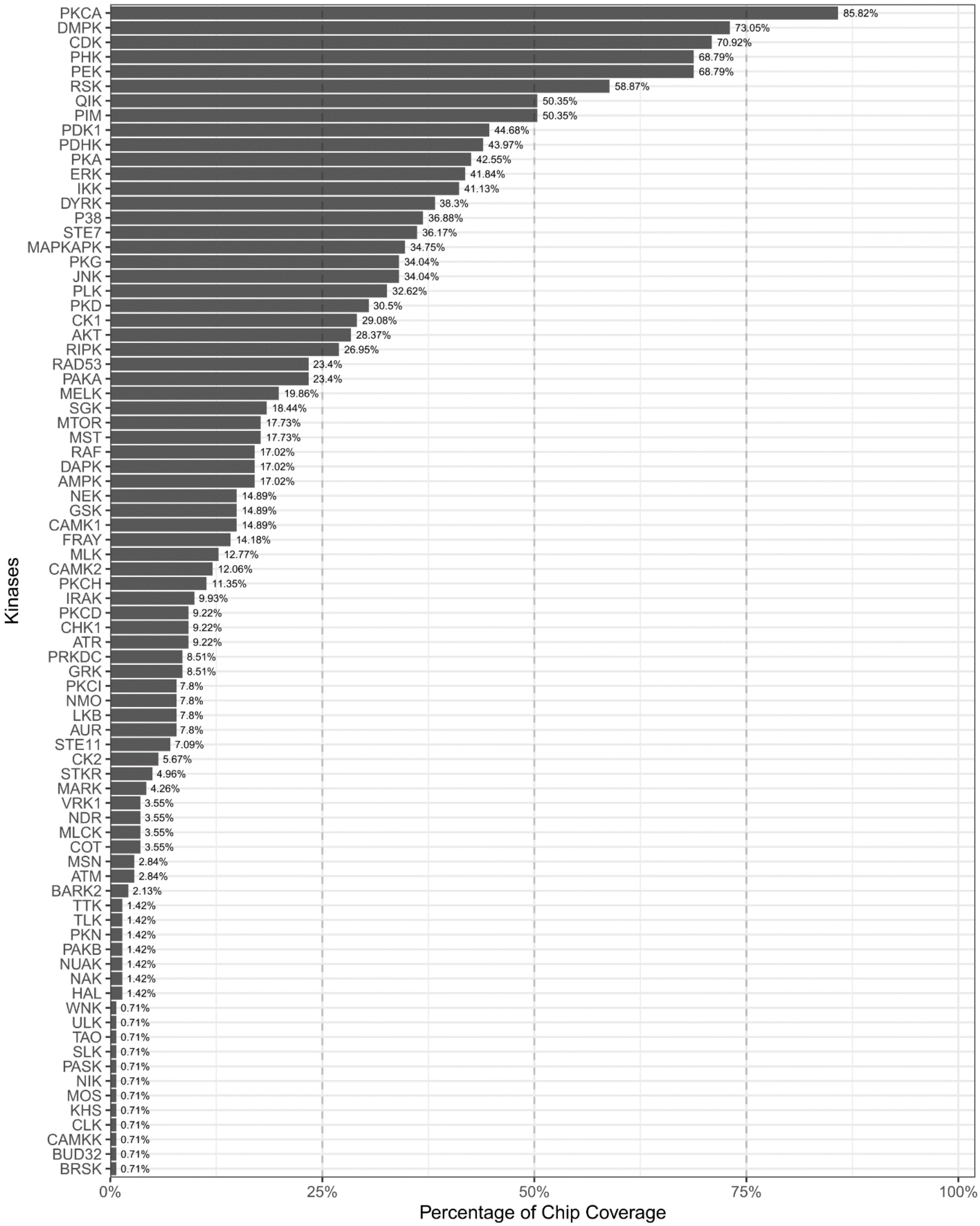

Fig. S8. Coverage of protein kinase families based on assignments of protein kinases to specific STK chip reporter peptides generated by the kinome resampling analyses (KRSA) package. The X-axis indicates the percentage of peptides on a chip that "map" to a kinase in a specific kinase family (Y-axis). There are 144 distinct reporter peptides on the STK chip.

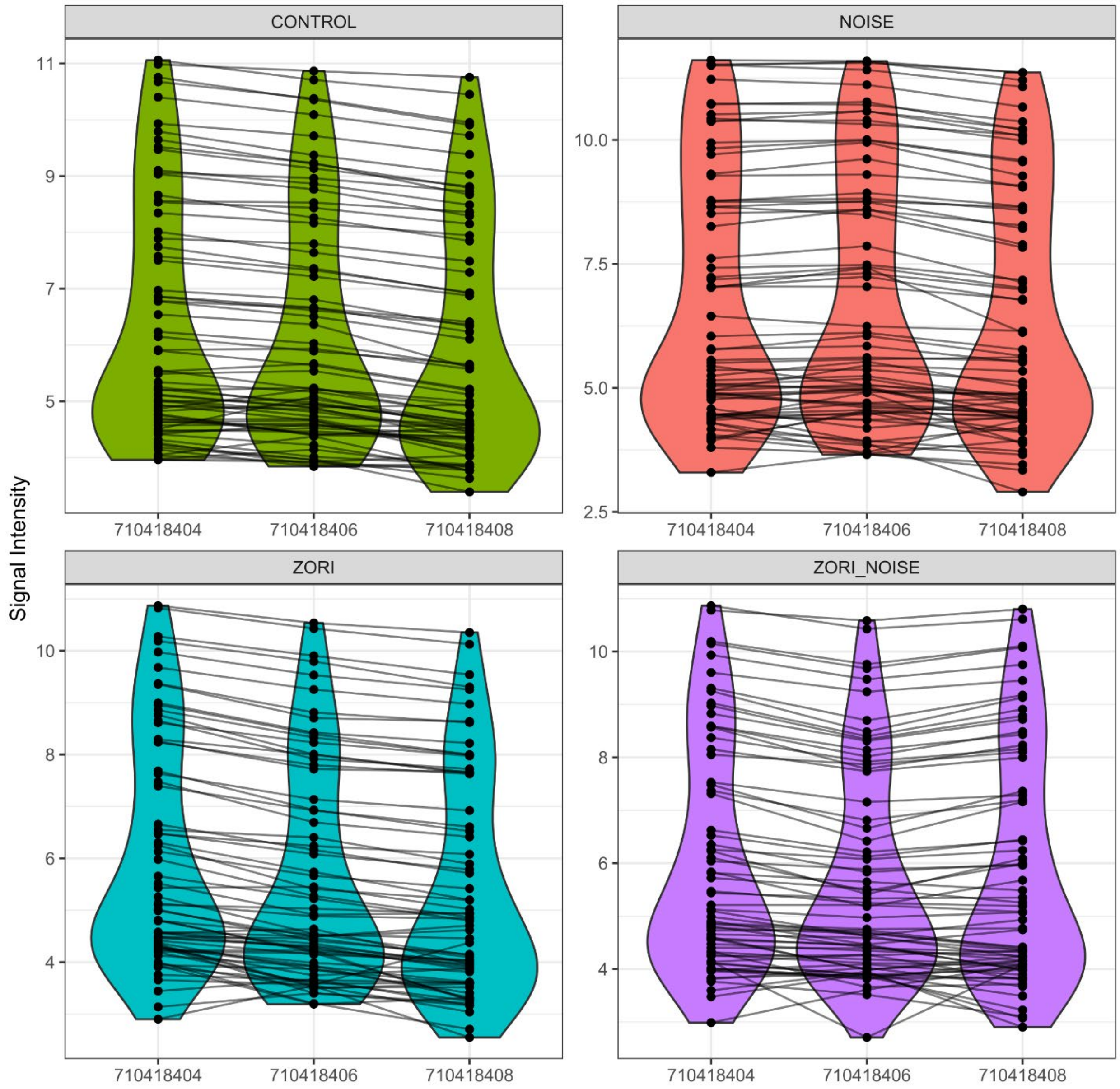

Fig. S9. Distribution of normalized signal intensities of reporter peptides for control, zorifertinib (Zori), noise, and zorifertinib plus noise groups across three STK chips. Each group was included on each of three chips, providing n = 3 technical replicates. Each chip has 4 wells.

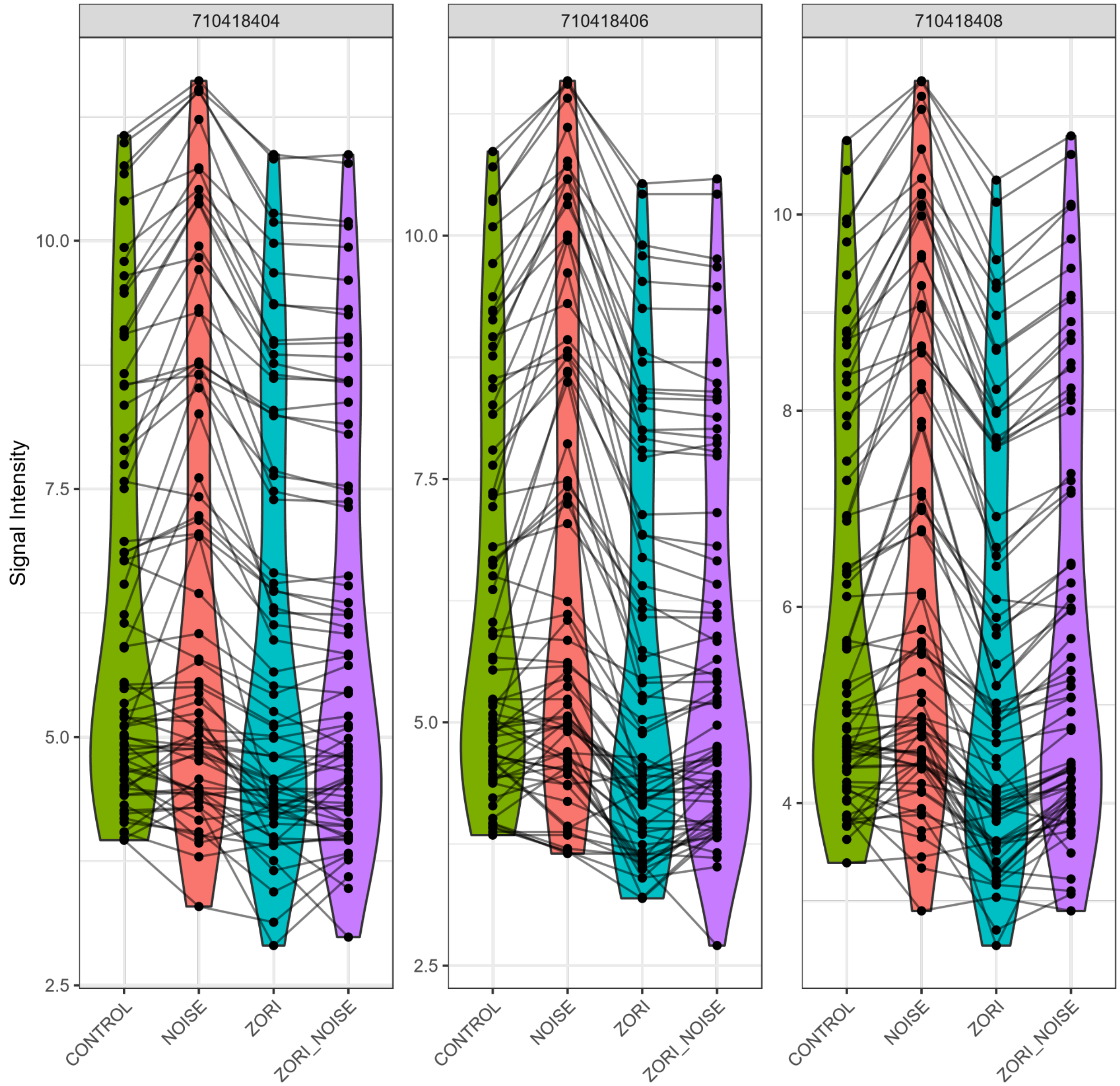

Fig. S10. Distribution of normalized signal intensities of reporter peptides for control, zorifertinib (Zori), noise, and zorifertinib plus noise groups between three STK chips. Each group was included on each of the three chips, providing n = 3 technical replicates. Each chip has 4 wells.

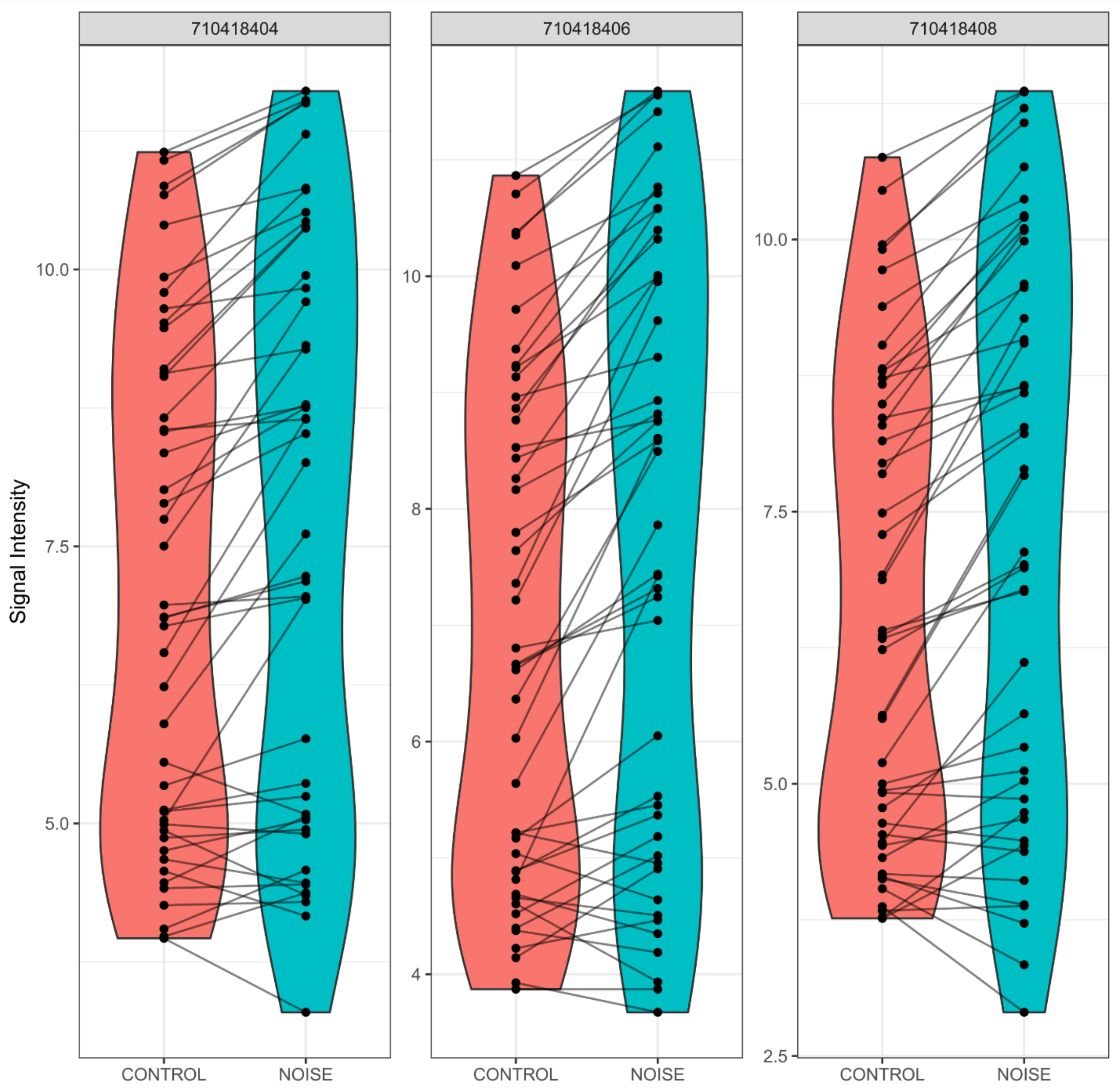

Fig. S11. Distribution of normalized signal intensities of reporter peptides for noise versus control groups only across three STK chips.

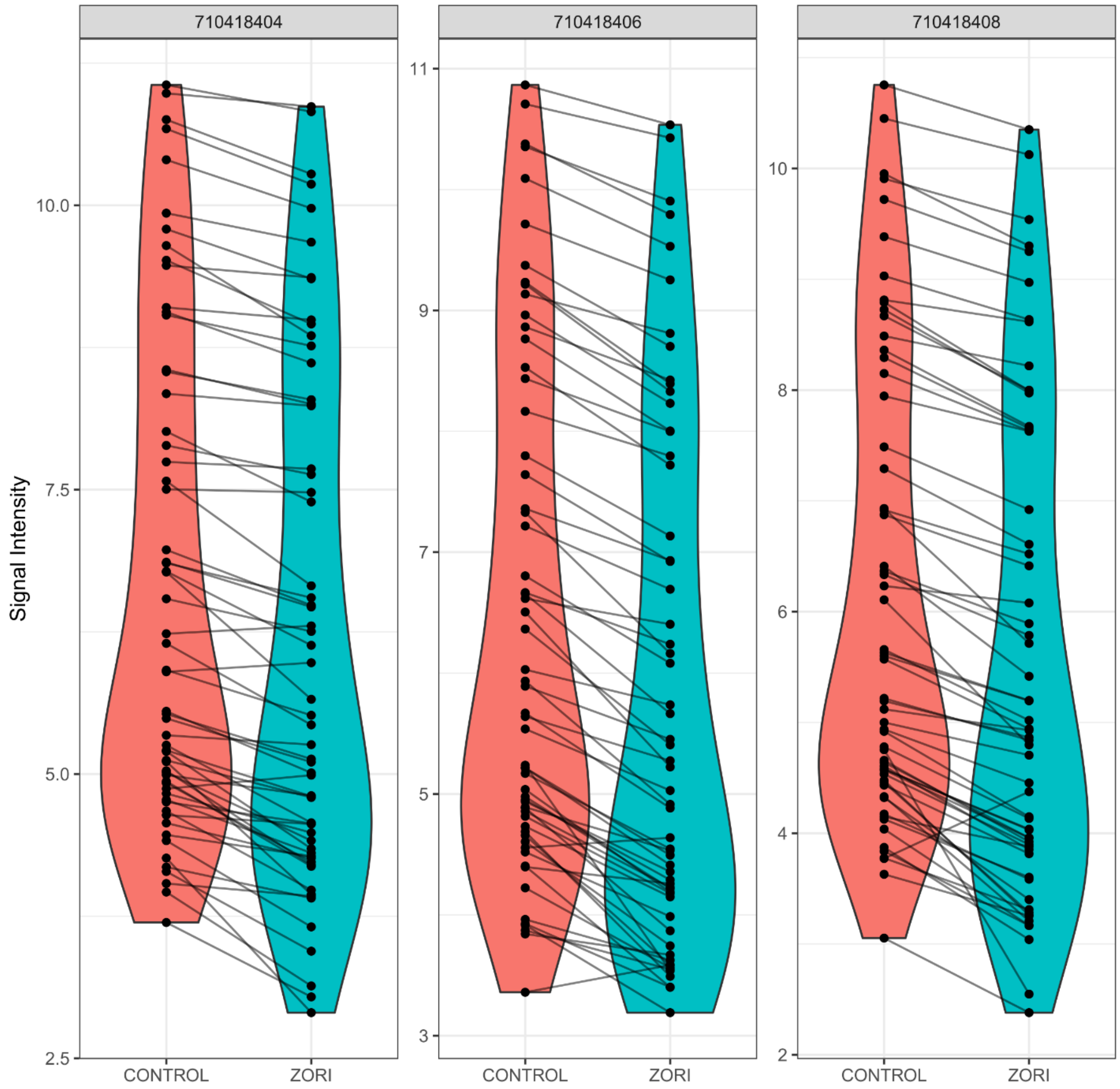

Fig. S12. Distribution of normalized signal intensities of reporter peptides for control versus zorifertinib (Zori) groups only across three STK chips.

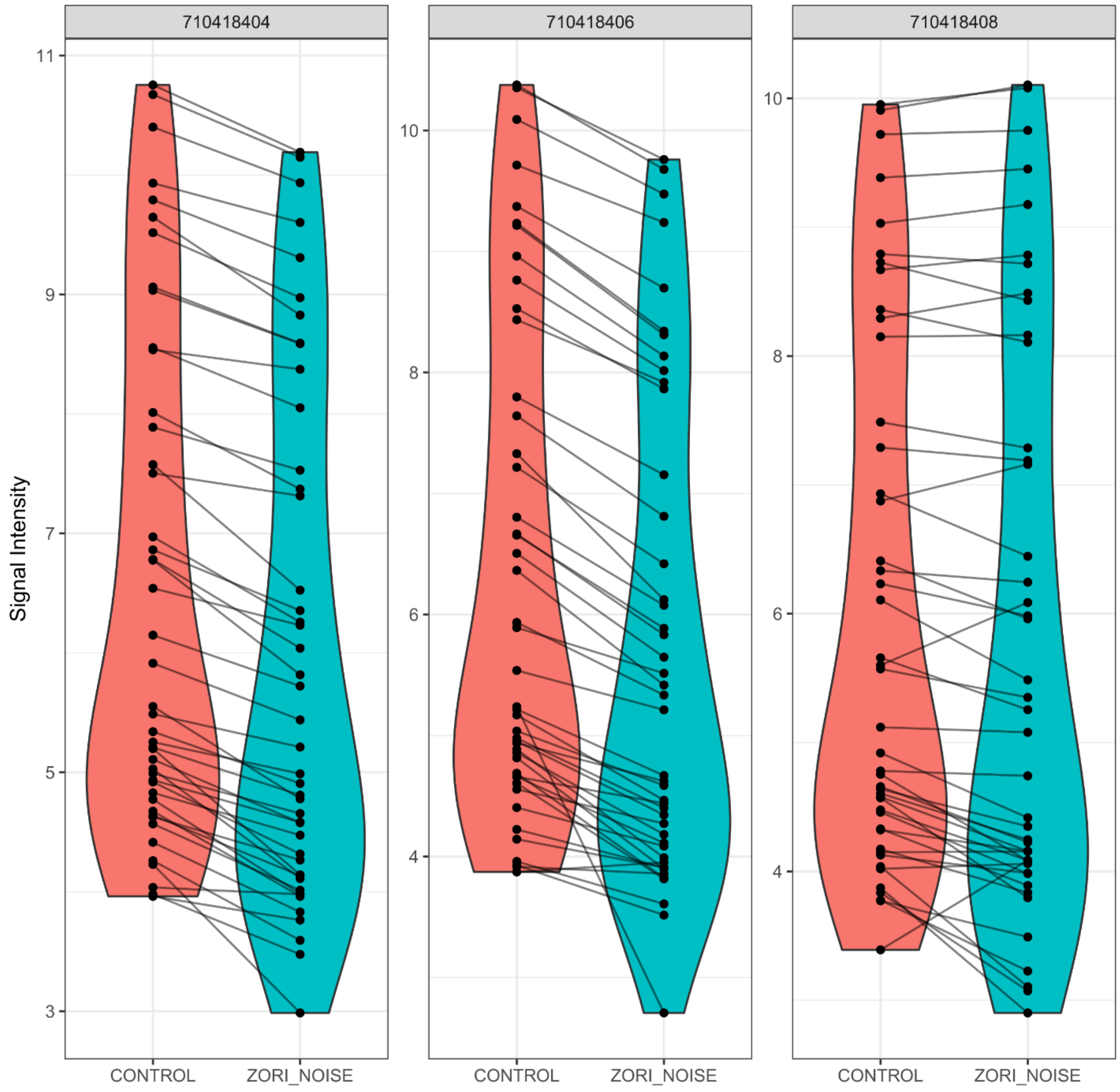

Fig. S13. Distribution of normalized signal intensities of reporter peptides for control versus zorifertinib (Zori) plus noise groups only across three STK chips.

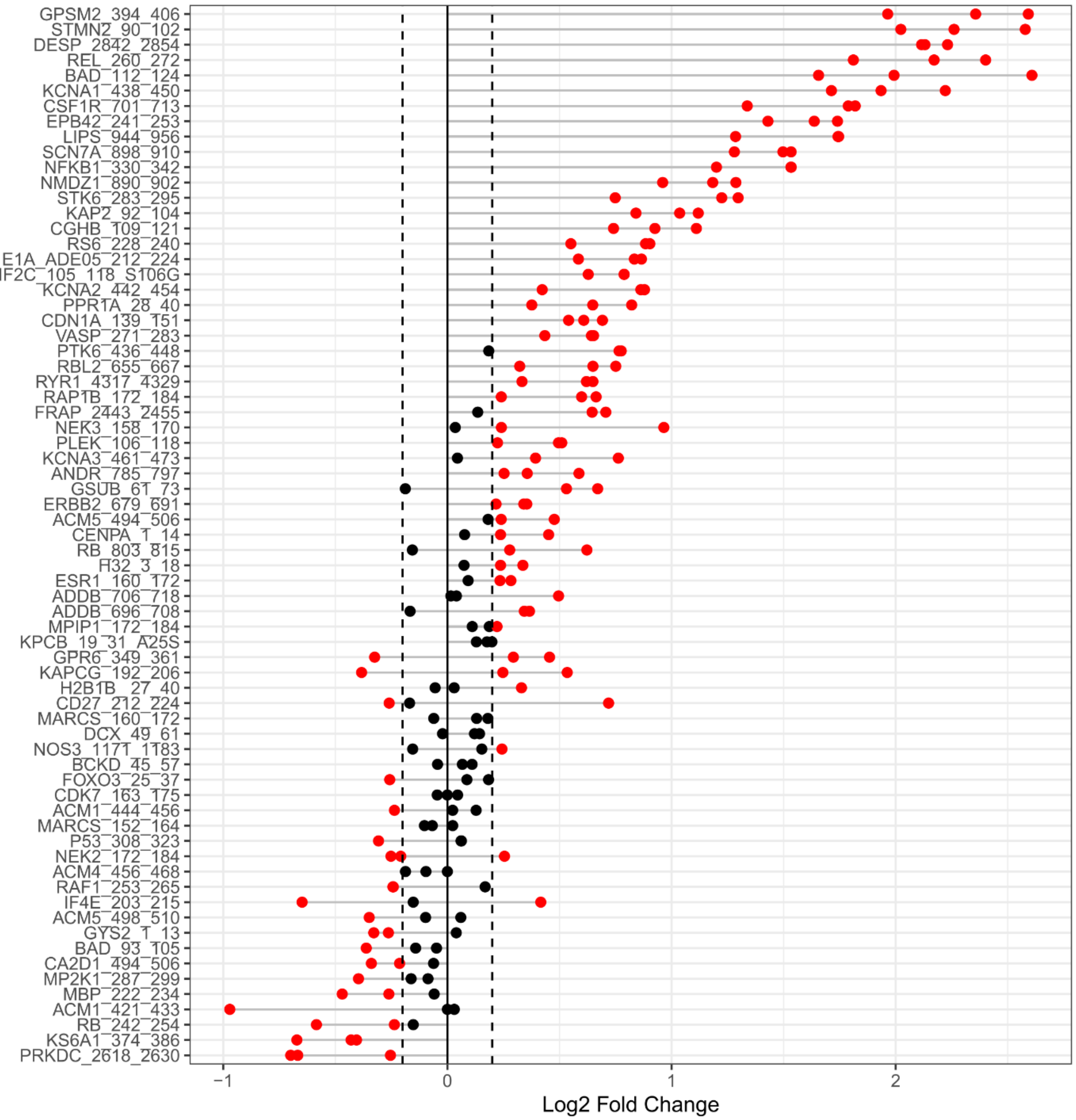

Fig. S14. Waterfall plot of Log2 fold change (X-axis) in phosphorylation of reporter peptides (Y-axis) in control vs noise cochlear homogenate from three STL chips. Each dot represents the value for that peptide from one of the three chips for the same comparison. Red dots are outside the threshold (±0.15) considered meaningful for changes in kinase activity.

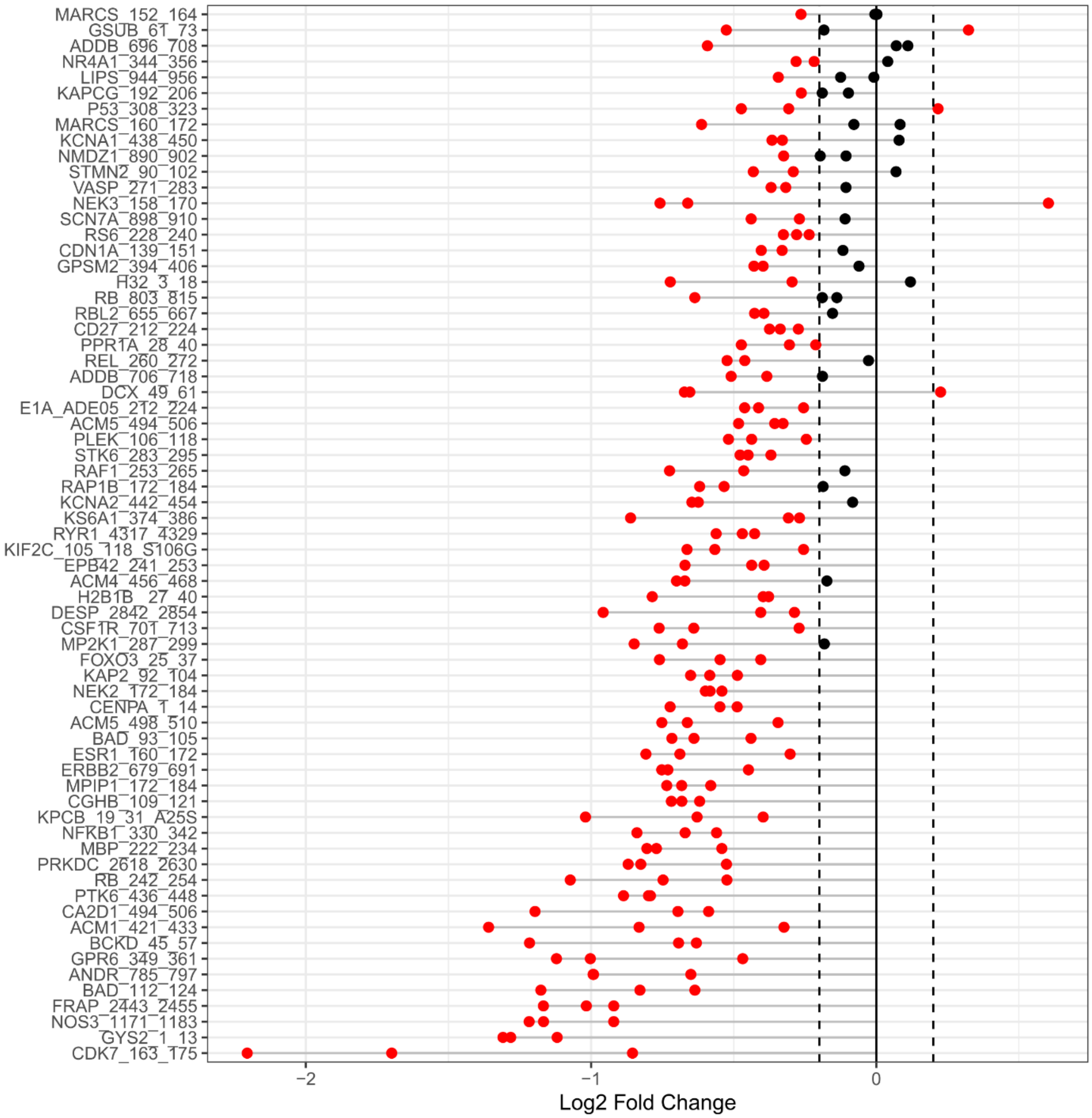

Fig. S15. Waterfall plot of Log2 fold change (X-axis) in phosphorylation of reporter peptides (Y-axis) in control vs zorifertinib (Zori) cochlear homogenate from three STL chips. Each dot represents the value for that peptide from one of the three chips for the same comparison. Red dots are outside the threshold (±0.15) considered meaningful for changes in kinase activity.

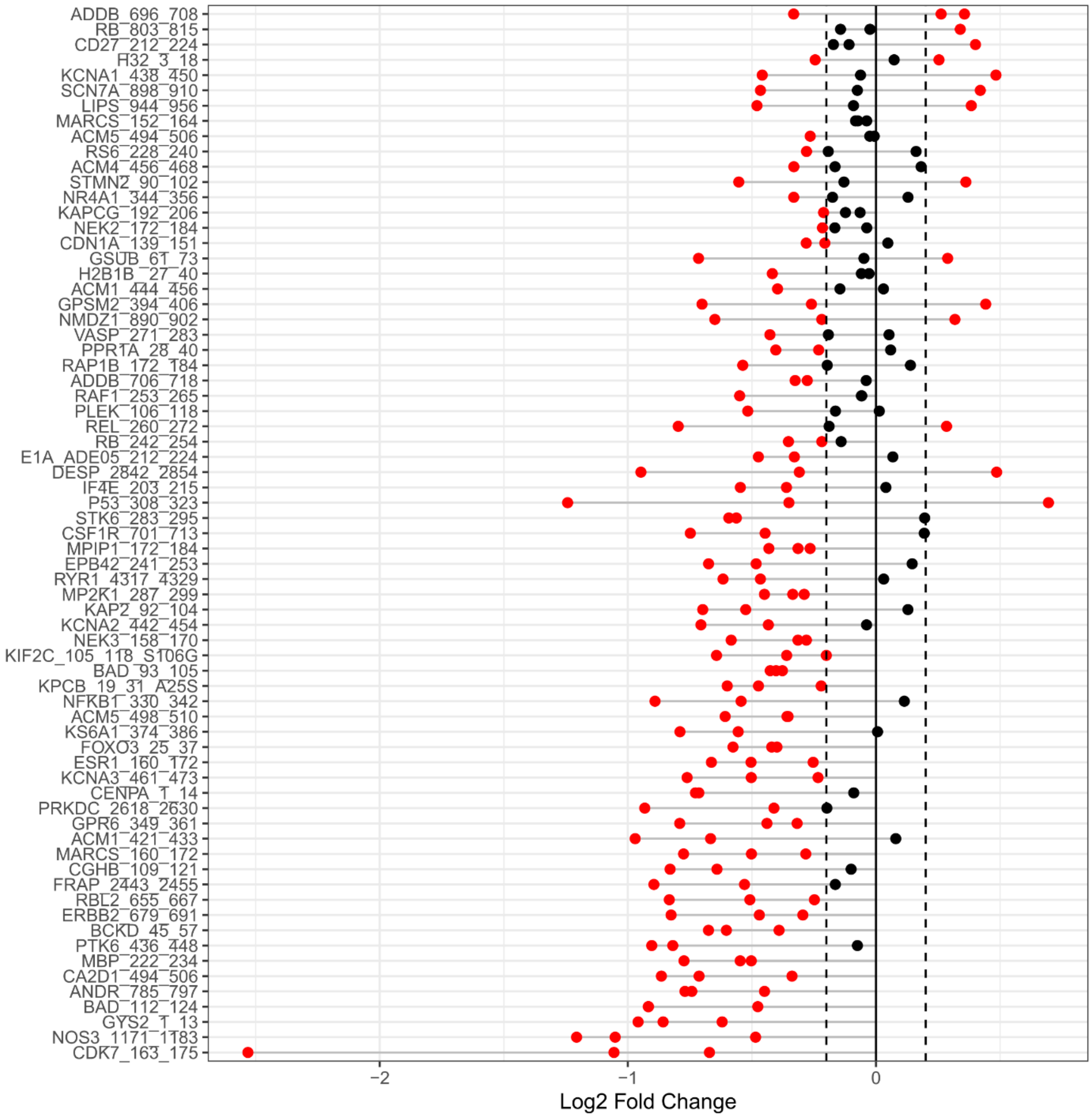

Fig. S16. Waterfall plot of Log2 fold change (X-axis) in phosphorylation of reporter peptides (Y-axis) in control vs zorifertinib (Zori) plus noise cochlear homogenate from three STL chips. Each dot represents the value for that peptide from one of the three chips for the same comparison. Red dots are outside the threshold (±0.15) considered meaningful for changes in kinase activity.

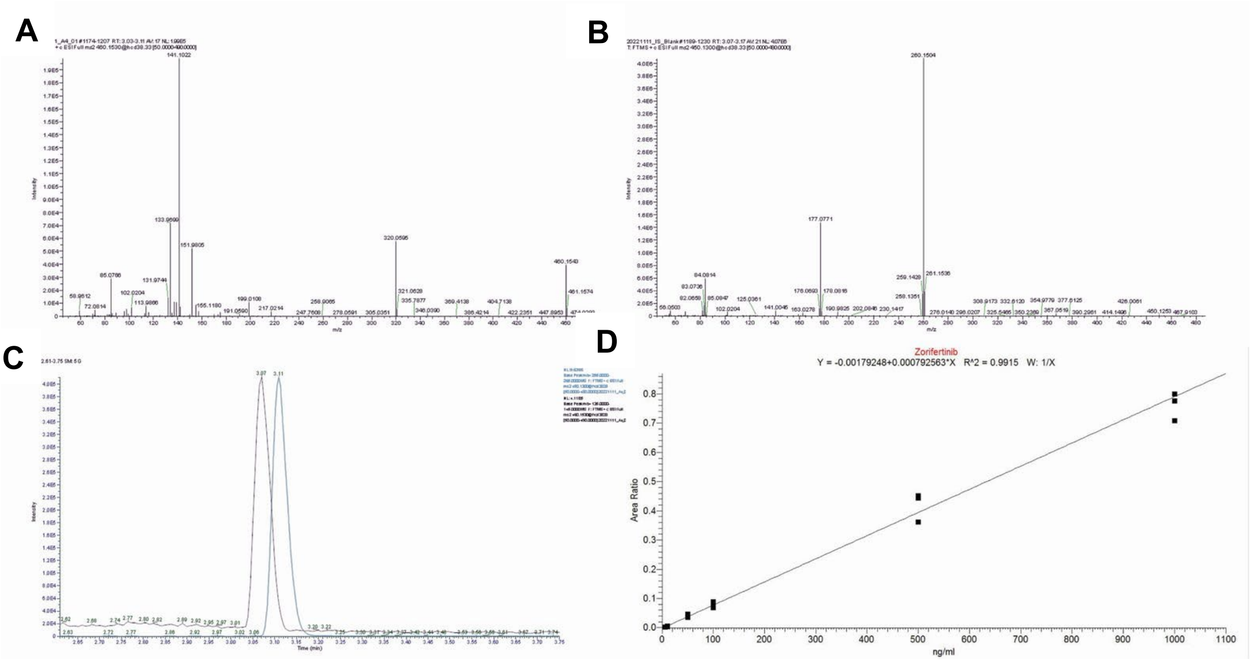

Fig. S17. (**A-B**) MS2 spectra of Zorifertinib (A) and IS- Crizotinib (B). (**C**) Sample chromatogram showing peaks for both zorifertinib (right) and IS (left). (**D**) Calibration curve linear regression.

Table S1. Biological Pathways and Candidate Drugs for NIHL. Differentially expressed genes, pathways and candidate drugs identified from the three transcriptomic datasets used for the study (related to Figure 1).
